# Multi‐Target Anti‐Obesity Potential of *Coula edulis* Nut Extract in High‐Fat Diet‐Induced Obesity in Rats

**DOI:** 10.1002/fsn3.72209

**Published:** 2026-08-13

**Authors:** Chidimma Emmanuel Ibeneme, Item Justin Atangwho, Godwin Eneji Egbung, Diana Ochuole Odey, Chinedum Ekeleme Martins, Uket Nta Obeten, Wilson Arong Obio, Louisiane Patrick Nangah, Emmanuel O. Ibeneme, Daniel Ejim Uti

**Affiliations:** ^1^ Department of Biochemistry, Faculty of Basic Medical Sciences University of Calabar Calabar Nigeria; ^2^ Department of Biochemistry, Faculty of Medicine and Pharmaceutical Sciences Kampala International University in Tanzania Dar es Salaam Tanzania; ^3^ Department of Biochemistry, Faculty of Science Southern Delta University Ozoro Nigeria; ^4^ Department of Chemistry/Biochemistry and Molecular Biology Alex Ekwueme Federal University Abakaliki Ebonyi State Nigeria; ^5^ Department of Chemical Sciences Top Faith University Uyo Akwa Ibom State Nigeria; ^6^ Department of Medical Bacteriology, Virology and Mycology, Faculty of Medical Laboratory Science University of Calabar Calabar Nigeria; ^7^ Department of Biochemistry, Research and Publications Kampala International University Kampala Uganda; ^8^ Department of Biochemistry, Faculty of Basic Medical Sciences, College of Medicine Federal University of Health Sciences Otukpo Benue State Nigeria

**Keywords:** adipogenesis, anti‐obesity, *Coula edulis* nut extract, high‐fat diet‐induced obesity, lipid metabolism, oxidative stress

## Abstract

Obesity is known to be associated with dyslipidaemia, cardiovascular disorders, oxidative stress, adipogenic and adipokine responses. The anti‐obesity effect of *Coula edulis* nut extract (CEE) was investigated in high‐fat‐diet (HFD) fed obese Wistar rats. After a four‐week HFD feeding, the rats received the nut extract *p.o*. for 5 weeks in a two‐batch design. Batch I: Normal Control (ND only) Obese Control (HFD only), Standard Control (HFD + 0.03 g/kg b.w. Orlistat) and extract‐treated groups 1 and 2 (HFD + CEE1g/kg b.w. and HFD + CEE2g/kg b.w., respectively). Batch II: Normal Control (ND only), Obese Control (ND only), Standard Control (ND + 0.03 g/kg b.w. Orlistat) and extract‐treated groups 1 & 2 (ND + CEE1g/kg b.w. and ND + CEE2g/kg b.w., respectively). During the 5‐week extract treatment, Batch I rats were fed HFD alongside, whereas the Batch II rats were fed normal diet (ND) instead. Weight gain, feed intake, anthropometric indicators, blood lipids, cardiovascular risk indices, pancreatic lipase activity, tissue lipid content, hepatic cholesterol biosynthesis, oxidative stress indices, expression of adipogenic/lipogenic genes and adipose histology were determined. *Coula edulis* extract was found to decrease weight gain, obesity indicators, lipogenesis, pancreatic lipase activity, cardiovascular risk factors, oxidative stress, and PPAR‐γ, C/EBP‐α and SREBP‐1c mRNA levels (*p* < 0). Together, the results indicate that *Coula edulis* nut extract could attenuate obesity by modulating lipid digestion, cholesterol synthesis, oxidative stress and adipogenic/lipogenic transcription factors.

## Graphical abstract

This study evaluated the multi‐target anti‐obesity effects of Coula edulis nut extract (CEE) in high‐fat diet‐induced obese Wistar rats. Following 4 weeks of obesity induction, rats received oral CEE at 1 or 2 g/kg body weight, or orlistat for 5 weeks under continued high‐fat diet or normal diet conditions. CEE reduced body weight gain, feed intake, anthropometric obesity indices, blood lipids, cardiovascular risk indices, pancreatic lipase activity, tissue lipid accumulation, and hepatic cholesterol biosynthesis. It also improved antioxidant status, reduced oxidative stress, suppressed adipose tissue hypertrophy, and downregulated PPAR‐γ, C/EBP‐α, and SREBP‐1c expression. These findings show that CEE attenuates obesity by inhibiting lipid digestion, cholesterol synthesis, oxidative damage, adipogenesis, and lipogenesis, thereby improving overall cardiometabolic health in obese rats.

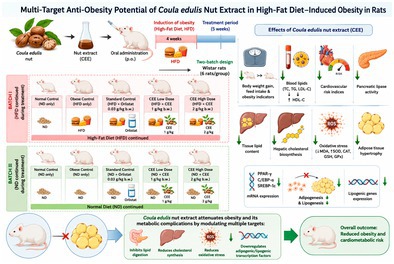

## 1 Introduction

Obesity and its associated comorbidities have become a serious global public health problem in the past few decades. According to the WHO, the number of obese men and women has tripled between 1975 and 2025 (Ahmed and Mohammed 2025). In 2022 alone, approximately 2.2 billion adults above 18 years, were overweight, and over 650 million were considered obese (World Health Organization 2025). In a Federal Secretariat Complex, Abuja, Nigeria, 64% of adult Nigerians (74% women and 57% men) were reported obese in 2014 (Odutola et al. 2019). In 2023, according to OECD report, Nigeria ranked among the top 10 countries globally with high obesity incidence—obesity prevalence of over 20 million individuals (Odutola et al. 2019; Overweight and Obesity 2023). Moreover, mortality and morbidity arising from overweight have increased tremendously (Overweight and Obesity 2025; Uti et al. 2026). Globalization and industrialization, i.e., increased consumption of energy‐dense meals such as pastries, ice cream, processed foods, refined carbohydrates and sweetened drinks are reported to be responsible for the increased rate of obesity, its comorbidities and mortality and morbidity (Umoru et al. 2025). Hence the dire need for radical prevention and treatment/intervention strategies.

Certain anti‐obesity agents have been in use since quite a long time (e.g., sibutramine, orlistat); however, there is a higher demand for newer and safer agents due to a variety of adverse effects such as bloating, oily spotting, diarrhea, fecal incontinence, flatulence, and dyspepsia (Bays et al. 2022). Lorcaserin, combination of phentermine and topiramate, and the combination of naltrexone/bupropion was recently approved for pharmacotherapy of obesity (Idrees et al. 2022). Again, the challenging clinical side effects and economic high cost associated with the use of these conventional anti‐obesity drugs, have led to the search for natural plant products, functional foods and dietary adjuncts that have minimal side effects, and are readily available and cost effective, but efficacious beneficial to obese patients.

Obesity largely involves aberration in lipid metabolism (Uti et al. 2026). Therapeutic interventions and lifestyle adjustment aim to reduce this aberration. One such intervention or strategy is sourcing bioactives and agents with the property of modulating the process of adipogenesis and lipolysis. The use of traditional foods and diets such as traditional herbs, functional diets, edible vegetables, nuts, and fruits have been found to be variable sources of these bioactive agents (Konstantinidi and Koutelidakis 2019). However, sound, basic, and rigorous scientific investigations to confirm and advocate the efficacies of these natural therapies and their therapeutic modes and mechanisms are absolutely necessary.

Nuts from *Coula edulis* can be eaten fermented, roasted, or boiled. In addition to being a source of flour and cooking oil, the nuts are utilized in dishes and combined with meats (Naccis et al. 2025). There are acetylenes in the dried bark that provide its anticancer properties (Naccis et al. 2025). In equatorial Africa, the bark powder is usually used for treatment of wounds and the bark infusion for curing anemia and increase of appetite (Kamdem et al. 2024). The decoction motive is used for curing oral infection and diarrhea while the stem bark for the treatment of ulcers (Kepawou et al. 2025). *Coula edulis* seed, which contained high concentration of flavonoid according to phytochemical analysis (Kepawou et al. 2025).

Moreover, from unpublished preliminary data obtained in our laboratory, ethanol extract of *Coula edulis* nut was found to modulate dyslipidemia in poloxamer‐induced hyperlipidemic rats by improving systemic effects on transport and storage lipids. On the strength of this observed anti‐hyperlipidemic action and its reported high flavonoid content, it became necessary to scientifically evaluate the body weight control action of the nut via its action in adipogenesis, and its effect on obesity‐associated low‐grade chronic inflammation and oxidative stress. And also, the need to understand the mode of action and molecular mechanism relating to lipid metabolism.

Therefore, the study evaluated the effect of *Coula edulis* nut extract on anthropometric parameters, serum lipid profiles, tissue lipid accumulation, intestinal lipid digestion through pancreatic lipase activity and hepatic cholesterol synthesis through HMG‐CoA reductase activity in high‐fat diet‐induced obesity in rats. Additionally, markers of oxidative stress, histological examination of adipose tissue and expression profiles of pivotal adipogenesis regulatory genes (PPAR γ, C/EBP α, SREBP‐1c), lipogenesis control gene (FAS), β‐oxidation of lipids (PPAR α) in the liver and the adipose and inflammation mediation genes (TNF‐α and adiponectin) were studied.

## 2 Materials and Methods

### 2.1 Preparation of Nut Extract

*Coula edulis* nuts collected from Akamkpa, Cross River State, Nigeria, were shelled, dried at 40°C, cooled, and ground into powder. The powdered sample was ethanol‐extracted using a Soxhlet apparatus. The solvent was removed by rotary evaporation and water‐bath concentration. From 5000 g starting material, 350 g extract was obtained, giving 7% yield (Al Juhaimi et al. 2018).

Percentage yield formula:

$$\begin{array}{c} \text{Percentage yield}\;\left(\%\right)\\ =\left(\text{Weight of dried extract}/\text{Weight of starting powdered}\;\mathrm{nut}\;\text{material}\right)\times 100 \end{array}$$

Using the reported values:

$$\text{Percentage yield}\;\left(\%\right)=\left(350\;g/5000\;g\right)\times 100=7\%$$

### 2.2 Formulation of High Fat Diet (HFD)

The high‐fat diet used in the induction of experimental obesity was formulated based on Yu et al. (2023) with slight modification. Briefly, the diet comprised 64% commercial rat pellet, 15% lard, 5% full‐cream milk powder, 5% sucrose, 5% egg yolk powder, 0.8% porcine bile salt, and 0.2% sodium chloride (Table 1). The HFD was prepared every other day by weighing out and reconstituting all ingredients (w/w). The proximate composition of the formulated HFD was determined alongside the normal rat chow (data not shown). The energy of the normal diet and high‐fat diet was calculated using the method described by Wang et al. (2019). In our formulation, the HFD was 28.8% higher in calorie content than the normal rat chow.

**TABLE 1:** Composition of high fat diet.

High fat diet components	Percentage composition
Normal diet	64
Lard	15
Sucrose	10
Egg yolk powder	5
Milk	5
Porcine bile salt	0.8
Sodium chloride	0.2
Total	100.0

### 2.3 Animal Handling

Male adult Wistar rats (140–180 g) were obtained and used for the study. For one (1) week, the study animals were permitted unlimited access to water and allowed to get used to the study environment and experimental diet (acclimatization). After acclimatization, the animals were divided into test and control groups and given care as indicated in the experimental design (Table 2). Animal experiments were reviewed and approved by the Faculty Animal Research Ethics Committee (FAREC‐FBMS), Faculty of Basic Medical Sciences, University of Calabar, Nigeria, with full ethical approval for the proposal titled “Molecular and in silico effects of Coula edulis nut extract on glucose metabolism in high fat diet obese Wistar rats.” Approval No.: 406BCM3225; Committee Ref.: FAREC/PA/UC/049; Date: 10/05/2023 (10 May 2023). The study was conducted in accordance with the ARRIVE (Animal Research: Reporting of in Vivo Experiments) 2.0 guidelines to ensure rigorous, transparent, and reproducible reporting. Key elements, including study design, sample size determination, blinding, experimental procedures, animal characteristics, outcome measures, and statistical analyses, were comprehensively described. Ethical considerations, animal welfare, and data reporting were also addressed in line with ARRIVE recommendations. See Tables S1–S3.

**TABLE 2:** Experimental design and treatment schedule.

S/N	Group	Number of rats	Treatment
1	Normal control (NC)	6	Distilled water
Batch I			
2	Obese control A (HFD + NT)	6	Distilled water
3	Standard control A (HFD + OR)	6	0.03 g/kg body weight of orlistat
4	Obese treated I A (HFD + CE1g)	6	1 g/kg body weight of CE extract
5	Obese treated II A (HFD + CE2g)	6	2 g/kg body weight of CE extract
Batch II			
6	Obese control B (ND + NT)	6	Distilled water
7	Standard control B (ND + OR)	6	0.03 g/kg body weight of orlistat
8	Obese treated I B (ND + CE1g)	6	1 g/kg body weight of CE extract
9	Obese treated II B (ND + CE2g)	6	2 g/kg body weight of CE extract

Abbreviations: CE, *Coula edulis*; CE1g, 1 g/kg of CE extract; CE2g, 2 g/kg of CE extract; HFD, High fat diet; ND, normal diet; NT, No treatment; OR, Orlistat.

### 2.4 Induction of Experimental Obesity

The experimental animals were fed a high‐fat diet for 4 weeks to induce obesity, with their body weight being monitored in the process. Rats having a Lee's index of 310 (Christoper et al. 2025), or a body weight increase ≥ 20% compared to the normal control group of rats fed standard rat chow only, were considered obese at the end of the 4‐week period (Shabbir et al. 2016). As indicated earlier, the high‐fat diet was prepared every other day to avoid spoilage.

### 2.5 Treatment Schedule/Protocol

Intervention with the *Coula edulis* extract at a 2‐dose level was carried out into two batches, i.e., the first batch continued with the high fat diet alongside extract administration while in the second batch, high fat diet was discontinued and replaced with normal rat chow during extract administration (*n* = 6 per group). The rationale behind this design is to determine the effectiveness of *Coula edulis* nut extract in body weight control in the presence or absence of high fat diet. Each of the batches consisted of 5 groups of 6 rats each, namely: Normal control (ND only), obese control (HFD or ND only), standard control (HFD +0.03 mg/kg b.w. Orlistat or ND +0.03 mg/kg b.w. Orlistat), treatment groups 1 and 2 (HFD +1 g/kg b.w. or 2 g/kg b.w. of CEE and ND + CEE1g or CEE 2 g/kg b.w.). The normal control group was common to both batches, making the fifth group for each batch. The complete treatment schedule is shown in Table 2 below.

### 2.6 Extract Administration and Sample Collection

The test groups received *Coula edulis* extract orally by gavage at 1 or 2 g/kg body weight, shown in Table 2. The doses were determined from preliminary anti‐hyperlipidaemic studies. Extract treatment commenced after obesity induction at week 5 and continued until week 9, lasting 5 weeks.

### 2.7 Dietary and Anthropometric Measurements

Body weight and feed/energy intake were measured weekly and calculated thus:

Percentage Yield:

$$\begin{array}{c} \text{Percentage yield}\;\left(\%\right)\\ =\left(\text{Weight of dried extract}/\text{Weight of starting powdered}\;\mathrm{nut}\;\text{material}\right)\times 100 \end{array}$$

Example: (350 g/5000 g) × 100 = 7%.

Percentage Change in Body Weight:

$$\begin{array}{c} \text{Percentage change in body weight}\;\left(\%\right)\\ =\left(\text{Final body weight}-\text{Initial body weight}\right)/\text{Initial body weight}\times 100 \end{array}$$

Daily Feed Intake:

$$\begin{array}{c} \text{Daily feed intake}\;\left(g/\mathrm{day}\right)\\ =\left(\text{Feed supplied}\;\left(g\right)-\text{Feed leftover}\;\left(g\right)\right)/\text{Number of days} \end{array}$$

Weekly Feed Intake:

$$\text{Weekly feed intake}\;\left(g/\text{week}\right)=\text{Daily feed intake}\;\left(g/\mathrm{day}\right)\times 7$$

Also, the amount of calories consumed per week was calculated as the product of average weekly feed intake and the caloric value of normal diet and high‐fat diet. The following nutritional parameters were calculated using the value of average feed intake and average caloric [17]:

Daily Energy Intake:

$$\begin{array}{c} \text{Daily energy intake}\;\left(\mathrm{kJ}/\mathrm{day}\right)\\ =\text{Daily feed intake}\;\left(g/\mathrm{day}\right)\times \text{Dietary metabolizable energy}\;\left(\mathrm{kJ}/g\right) \end{array}$$

Weekly Energy Intake:

$$\text{Weekly energy intake}\;\left(\mathrm{kJ}/\text{week}\right)=\text{Daily energy intake}\;\left(\mathrm{kJ}/\mathrm{day}\right)\times 7$$

### 2.8 Sample Collection

At the end of treatment in week 5, the rats were fasted overnight and anesthetized with ketamine to reduce pain (15 mg/kg b.w.). Blood was obtained by cardiac puncture into non‐heparinized tubes, allowed to stand for 2 h, and afterwards, centrifuged at 2000 ***g*** for 15 min. After centrifugation, the serum was collected and stored at −4°C. Heart, kidneys, and white adipose tissues were excised, blotted on a capillary paper, weighed, and normalized to body weight. Thereafter, the tissues were stored frozen and a portion of each fixed in 10% formalin for histological studies.

### 2.9 Biochemical Assays in Serum

#### 2.9.1 Determination of Serum Lipid Profile

Serum total cholesterol, HDL‐c, and triacylglycerol were measured using Fortress Diagnostics kits according to the manufacturer's instructions. LDL‐c and VLDL‐c were estimated indirectly from other measured lipid indicators values using standard calculation formulas: $\mathrm{LDL}-c=\mathrm{TC}-\left[\mathrm{HDL}-c+\mathrm{TAG}/5\right]$, and $\text{VLDL}-c=\mathrm{TC}-\left[\mathrm{HDL}-c+\mathrm{LDL}-c\right]$ (Uti et al. 2020a).

#### 2.9.2 Determination of Serum Pancreatic Lipase Activity

Serum pancreatic lipase activity was determined using a kinetic photometric method (Fossati et al. 1992). The reaction was triggered by the addition of the serum (or calibrator/control), immediate mixing, and the recording of absorbance in kinetic mode at the recommended wavelength for methylresorufin (approximately 580 nm). Measurements were made at fixed intervals for 2–5 min to check the linearity. Lipase activity (U/L) was determined by the kit‐provided factor. Quality controls were included in each run, and a few samples with large lipemia or hemolyzing were not considered to minimize interference.

#### 2.9.3 Determination of Atherogenic Index (AI), Atherogenic Coefficient (AC), and Coronary Risk Ratio (CRR)

Atherogenic index (AI) was determined using the formula earlier used by Mba et al. (2026):

$$\text{Atherogenic index}\;\left(\mathrm{AI}\right)=\frac{\mathrm{LDL}-c}{\mathrm{HDL}-c}$$

Atherogenic coefficient (AC) was determined using the formula:

$$\text{Atherogenic coefficient}\;\left(\mathrm{AC}\right)=\frac{\mathrm{TC}-\mathrm{HDL}-c}{\mathrm{HDL}-c}$$

Coronary risk ratio (CRR) was determined using the formula:

$$\text{Cardiac risk ratio}\;\left(\mathrm{CRR}\right)=\frac{\mathrm{TC}}{\mathrm{HDL}-c}$$

### 2.10 Evaluation of Tissue Lipids

#### 2.10.1 Preparation of Tissue Homogenates for Evaluation of Lipids

Tissue lipids were extracted by homogenizing samples in chloroform–methanol, followed by chilling, centrifugation, washing with potassium chloride, re‐centrifugation, and storage of the lipid‐rich chloroform phase at −20°C (Uti et al. 2020b).

#### 2.10.2 Estimation of Total Lipids in the Homogenates

Folch method was used in the determination of total tissue lipids (Eggers and Schwudke 2016). To plain tubes pre‐weighed, 1 mL of the lipid extract was pipetted and allowed to evaporate to dryness. Afterwards, the tubes were reweighed, allowed to cool, and the difference in weight between the empty tubes and the tubes plus the extract was recorded as total lipids. The result was expressed as mg/g of the weight of the organ.

#### 2.10.3 Estimation of Total Cholesterol, Phospholipids, Triacylglycerol, and Hepatic 3‐Hydroxyl‐3‐Methylglutaryl‐CoA (HMG‐CoA) Reductase Activity in the Homogenates

Total cholesterol in the tissue homogenate was determined using Folch method (Folch et al. 1957). Tissue phospholipids and triacylglycerol were measured following the procedure presented by Connerty et al. (1961), while hepatic HMG‐CoA reductase activity was estimated from the HMG‐CoA/mevalonate ratio. Uti et al. (2020a) following the earlier method of Rao and Ramakrishnan (1975).

#### 2.10.4 Estimation of the Magnitude of Cholesterol Synthesis (MCS)

Hepatic cholesterol synthesis was estimated from wet liver weight and TMAR, expressed as percentage mevalonic acid synthesis (Uti et al. 2020a):

$$\text{TMAR}=\frac{\mathrm{HMG}-\mathrm{CoA}\;\text{concentration}}{\text{Mevalonate concentration}}$$

$$\mathrm{MCS}\;\left(\%/g\;\text{liver weight}\right)=\frac{\mathrm{Wet}\;\text{liver weight}\;\left(g\right)}{\text{TMAR}}\times 100$$

#### 2.10.5 Percentage Reduction in HMG‐CoA Reductase Activity

HMG‐CoA reductase activity reduction was calculated as 100 minus magnitude of cholesterol synthesis (Uti et al. 2020a).

### 2.11 Evaluation of Oxidative Stress and Lipid Peroxidation Indicators

#### 2.11.1 Preparation of Tissue Homogenate for Oxidative Stress Assay

For measurement of oxidative stress, 0.2 g of tissue was homogenized in 1.8 mL of pH 7.2 buffer, which contained 5 mM HEPES, 125 mM mannitol, 125 mM sucrose and 1 mM EGTA. The mixture was then centrifuged at 3000 rpm for 10 min and the supernatant was kept at −20°C until analysis.

#### 2.11.2 Determination of Superoxide Dismutase, Glutathione Peroxidase, and Reduced Glutathione Activity

The method previously described by Engelbrecht et al. (2023) in the determination of superoxide dismutase (SOD) activity was used. In this method, there is competition for O^2−^ by SOD and pyrogallol. SOD activity was calculated thus:

The SOD activity was calculated from the percentage inhibition of pyrogallol autoxidation as follows:

$$\begin{array}{c} \text{Percentage inhibition}\;\left(\%\right)\\ =\left[\left(\mathrm{\Delta A}\;\text{blank}/\min-\mathrm{\Delta A}\;\text{sample}/\min\right)/\left(\mathrm{\Delta A}\;\text{blank}/\min\right)\right]\times 100 \end{array}$$

where absorbance was read at 420 nm.

$$\mathrm{SOD}\;\text{units}=\text{Percentage inhibition}/\left(100-\text{Percentage inhibition}\right)$$

The enzyme activity was then corrected for dilution:

$$\mathrm{SOD}\;\text{activity}\;\left(\text{units}/\mathrm{mL}\right)=\mathrm{SOD}\;\text{units}\times \text{dilution factor}$$

Finally, activity was normalized to protein concentration:

$$\begin{array}{c} \mathrm{SOD}\;\text{activity}\;\left(\text{units}/\mathrm{mg}\;\text{protein}\right)\\ =\mathrm{SOD}\;\text{activity}\;\left(\text{units}/\mathrm{mL}\right)/\text{protein concentration}\;\left(\mathrm{mg}/\mathrm{mL}\right) \end{array}$$

Glutathione peroxidase (GPx) activity was measured using the procedure first outlined by Ellman (1959). This protocol produces a restored 2‐Nitro‐5‐Thiobenzoic Acid (NTB) and a mixed disulfide after oxidation of a free ART through oxidation with 5,5′‐Dithiobis (2‐Nitrobenzoic Acid) (DTNB). Calculation: Molar extinction coefficient for NTB (14.15 mM‐1 cm‐1) was used to calculate the concentration of GSH in the sample. Absorbance/14.15 = GPx (mM). GPx (mM) × 10^−3^ = final value. Reduced glutathione level was determined using a previously described method by Ellman (1959).

#### 2.11.3 Estimation of Extent of Lipid Peroxidation Using TBARS‐MDA Reagent

A previously described method by Buege and Aust (1978) was used in the estimation of the extent of lipid peroxidation. A solution of 4.0 mm M Thiobarbituric acid (TBA) in glacial acetic acid was prepared to prepare. In order to prepare this, 57.66 mg of TBA is dissolved in 100 mL of glacial acetic acid. A new solution of TBA was prepared every day to make for accurate data. The 4.0 mM TBARS reagent is then diluted using a 75:25 mix of distilled water and TBARS reagent to make a 1.0 mM working reagent.

In the assay, 50 mL of the sample under investigation is mixed with 1 mL of the TBARS reagent. This solution is incubated at 95°C, 15 min, until it turns yellow, which means that TBARS complex is formed. This is followed by heating and the mixture is cooled in an ice bath and centrifuged at 1000 rpm in the ice bath in order to separate the supernatant. The supernatant is measured at 535 nm of the absorbance. The TBARS concentration is measured as M/mg protein. The extinction coefficient (E) of the MDA TBA complex at 535 nm, which is 1.56 × 105M −1 cm^−1^, is used to determine the TBARS concentration (i.e., MDA).

$$\mathrm{NB}:\text{Concentration}\;\left(M\right)=\frac{\text{Absorbance}}{\varepsilon }$$

where *Ɛ* is molar extinction coefficient, usually 1.56 × 10^5^ M^−1^ cm^−1^.

### 2.12 Gene Expression Analyses

#### 2.12.1 Tissue Homogenization and RNA Extraction

Homogenization of the liver tissue and RNA extraction were carried out as in our previous report (Atangwho et al. 2014). The first step was the homogenization of 50 mg of liver tissue in 500 mL of Trizol reagent. Then, 100 mL of chloroform was sprayed on the mixture and allowed to shake vigorously within a period of 30 s before letting it rest after a period of 10 min at room temperature. The process made the separation of the homogenate into three layers possible upon centrifugation at 11,300 rpm, 15 min later, and the topmost layer was made of soluble RNA. This supernatant was carefully aspirated into fresh tubes where 0.25 mL of cold isopropyl alcohol was put to precipitate the RNA. After this, vortexing the mixture was followed by another 10 min incubation at room temperature and centrifugation at 12,000 rpm in 10 min to isolate the RNA pellets.

A sterile tube was prepared to purify the RNA pellets and this was done by adding 500 mL of cold 75% ethanol, inverting, and centrifuging them after 5 min at 9100 rpm. The supernatant was discarded with caution and the remaining pellet dried under a period of about 10 to 15 min by a Centrivap. The resulting pellets of RNA were re‐suspended in 50 mL of RNase‐free water and stored at the temperature of −20°C and thus made the purified RNA extract a viable input in further gene‐expression experiments.

#### 2.12.2 Determination of Purity and Concentration of RNA

Nano‐spectrophotometer (Nano drop 2000, Thermo Fisher Scientific Nano Drop 2000) was used in the evaluation of the concentration and purity of the extracted RNA at 260/280 nm. A Nano drop of the RNA extract on the Nano‐spectrophotometer measured directly the RNA concentration and purity factor, which is a ratio of the extract absorbance at 260–280 nm values ≥ 1.8 and ≤ 2.2 are considered appropriate for pure extract (Umoru et al. 2025).

#### 2.12.3 Synthesis of Complementary DNA (cDNA)

The isolated total RNA was reverse transcribed using the Transgen EasyScript one‐step RT‐PCR supermix (Beijing TransGen Biotech Co. Ltd., Beijing, China) following the manufacturer's guidelines.

#### 2.12.4 Amplification by Conventional Polymerase Chain Reaction (PCR)

The conventional amplification of the synthesized cDNA was performed using the Transgen EasyScript one‐step RT‐PCR supermix (Components‐ EasyScript one‐step Enzyme Mix‐ 80 μL, 2 × One‐Step Reaction Mix‐ 2 × 1 mL, RNase‐free Water‐ 2 × 1 mL) (Beijing TransGen Biotech Co. Ltd., Beijing, China). The total volume (20 μL) for each PCR reaction consisted of the following: 0.4 μL each of the forward and reverse primers (Table 3), 3.8 μL of nuclease‐free water, 0.4 μL Enzyme Mix, 10 μL reaction mix, and 5 μL RNA sample. The PCR reaction was performed using the Bio‐rad c1000 touch thermo‐cycler with the cycle parameters shown in Table 4.

**TABLE 3:** The primer sequences of the genes studied including the house‐keeping/reference gene.

Gene	Primer	Sequence (5′‐3′)	Length	Tm (°C)	GC (%)
PPARγ	Primer F	GGG TGC AGC GCT AAA TTC AT	20	56.4	50.0
	Primer R	CCA AAC CTG ATG GCA TTG TCA G	22	56.6	50.0
SREBP 1c	Primer F	TCT TGA CCG ACA TCG AAG ACA T	22	53.0	45.5
	Primer R	GTT CCT GAG AAG CCG GAA GG	20	55.9	60.0
C/EBP α	Primer F	CGC GGT GGC GGA ACG	15	52.9	80.0
	Primer R	ATC TTC ACA TGT ACC TGC GCC	21	54.4	52.4
B‐Actin	Primer F	CCC GCG AGT ACA ACC TTC TT	20	53.8	55.0
	Primer R	TAC CCA CCA TCA CAC CCT GG	20	55.9	60.0

**TABLE 4:** Outline of thermal cycling conditions.

Temperature	Time	Function
45°C	30 min	cDNA synthesis
93°C	1 min	Reverse transcriptase inactivation
94°C	30 s	Initial Denaturation
50°C	30 s	Annealing Temperature
72°C	1 min	Extension
72°C	10 min	Final Extension
4°C	Infinite	Infinite hold

#### 2.12.5 Gel Electrophoresis

RT‐PCR‐amplified cDNA was detected by agarose gel electrophoresis using 1.5% agarose in TBE buffer, stained with ethidium bromide, and cast into wells (Atangwho et al. 2014). TBE buffer (1.0%) was added to cover the gel after mounting it in an electrophoretic tank. The 5 μL of PCR solutions were applied gently onto the wells alongside the blank and molecular weight marker, and the electrophoresis ran for 30 min at 95 V and 400 mA. Thereafter, a UV trans‐illuminator was used to examine the gels, and band concentration was determined using the Image J software (NCBI).

#### 2.12.6 Estimation of Amplified cDNA

Image J software was used to estimate the concentration/intensity of the bands on the electropherogram which represent the quality of cDNA amplified per sample, directly proportional to the extent of expression of the gene. The data obtained was the expression of the gene relative to the house‐keeping gene (β‐actin).

### 2.13 Histological Assay (Hematoxylin and Eosin Staining)

Tissues previously fixed in 10% formalin were sliced to 1 cm thick, with a microtome and put in cassettes. The cassettes were processed in a tissue processor, which consisted of automatic impregnation for 14 h, xylene and wax cleaning, and alcohol dehydration. Melted paraffin was used to embed the cassettes and the assemblies were cooled to create paraffin blocks. Block trimming was performed and the material was sectioned using a microtome. Prior to mounting with DPX for microscopic examination, the sections were stained with hematoxylin and eosin (H&E) (Huang et al. 2006).

### 2.14 Statistical Analysis

Data were analyzed using ANOVA, Tukey's test, and Pearson correlation in GraphPad Prism, with significance set at *p* < 0.05.

## 3 Results

### 3.1 Diet Composition and Diet Intake

The proximate composition of the formulated high‐fat diet and the normal diet is presented in Table 5. Energy contribution by nutrient was estimated in both high‐fat diet (HFD) and normal diet (ND). Whereas the carbohydrate and fat contents of the high‐fat diet were higher than that of the normal diet by 20.6% and 63.7%, respectively, the protein was rather higher in the normal diet compared to the high‐fat diet by 36.5%. This translated to total metabolizable energy content of the high‐fat diet being 516.89 KJ/100 g (28.8%) higher than that of the normal diet (*p* < 0.05). Also, the food consumption pattern and energy intake during the obesity induction phase are shown in Table 6. There was no significant difference in food intake of all the high‐fat diet fed groups compared with the normal control, except HFD 6 that had the highest food consumption in week 4 (*p* < 0.05).

**TABLE 5:** Proximate composition and caloric value of the high‐fat diet used in this study.

Proximate parameter	High fat diet (per 100 g)	Calorie (KJ/100 g)	Normal diet (per 100 g)	Calorie (KJ/100 g)	Nutrient difference	Calorie difference
Moisture Content (%)	5.87 ± 0.10		9.63 ± 0.36			
Fat (%)	20.10 ± 0.83	743.70 ± 0.9^a^	7.29 ± 0.40	269.73 ± 0.5^b^	12.81 ± 0.65	473.97 ± 0.5
Ash (%)	3.21 ± 0.18		10.18 ± 0.85			
Crude Fiber (%)	9.19 ± 0.87		13.78 ± 1.00			
Protein (%)	13.06 ± 0.87	222.02 ± 0.85^bc^	20.57 ± 1.04	349.69 ± 0.98^d^	−7.51 ± 0.89	−127.67 ± 0.9
Carbobydrate (%)	48.58 ± 1.15	825.86 ± 0.97^e^	38.55 ± 1.27	655.30 ± 1.32^f^	10.03 ± 1.07	170.56 ± 1.02
Total (100%)	100.01 ± 4	1791.58 ± 2.72^g^	100 ± 4.92	1274.72 ± 2.8^h^		516.86 ± 2.42

*Note:* Groups with similar alphabets = no significant difference (*p* > 0.05). Values are expressed as mean ± SD, *n* = 3.

**TABLE 6:** Mean daily diet and energy intakes during obesity induction phase (week 1 to week 4).

Groups	Treatment	Week 1		Week 2		Week 3		Week 4	
		Diet intake (g)	Energy intake (KJ)	Diet intake (g)	Energy intake (KJ)	Diet intake (g)	Energy intake (KJ)	Diet intake (g)	Energy intake (KJ)
1	NC	67.45 ± 2.17^a^	859.86 ± 27.68^a^	64.80 ± 1.70^ab^	767.07 ± 62.33^a^	59.52 ± 3.18^a^	704.49 ± 65.93^a^	61.41 ± 2.74^a^	782.74 ± 34.97^a^
2	HFD 1	73.86 ± 4.10^a^	1323.23 ± 73.37^b^	65.66 ± 4.88^ab^	1092.26 ± 116.65^ab^	58.75 ± 3.64^a^	977.37 ± 96.41^b^	55.78 ± 3.41^a^	999.39 ± 61.11^b^
3	HFD 2	66.26 ± 4.25^a^	1187.15 ± 76.09^b^	64.63 ± 6.53^ab^	1075.26 ± 136.32^ab^	58.25 ± 2.99^a^	969.09 ± 89.50^ab^	56.01 ± 2.92^a^	1003.46 ± 52.29^b^
4	HFD 3	64.45 ± 4.10^a^	1154.67 ± 73.51^b^	56.91 ± 3.59^a^	946.81 ± 94.13^ab^	55.84 ± 2.40^a^	929.01 ± 81.83^ab^	58.27 ± 2.28^a^	1043.89 ± 40.89^b^
5	HFD 4	67.81 ± 5.65^a^	1214.82 ± 101.30^b^	71.88 ± 3.77^b^	1195.82 ± 111.26^b^	54.17 ± 2.62^a^	901.20 ± 81.85^ab^	60.19 ± 1.97^a^	1078.30 ± 35.29^b^
6	HFD 5	69.18 ± 4.47^a^	1239.47 ± 80.06^b^	67.52 ± 4.27^ab^	1123.19 ± 111.72^b^	54.45 ± 2.26^a^	905.90 ± 79.15^ab^	61.18 ± 2.43^a^	1096.17 ± 43.52^b^
7	HFD 6	70.06 ± 2.68^a^	1255.17 ± 47.99^b^	72.33 ± 3.11^b^	1203.31 ± 105.99^b^	62.15 ± 1.77^a^	1033.97 ± 84.77^b^	69.52 ± 1.39^b^	1245.47 ± 24.88^b^
8	HFD 7	67.06 ± 4.59^a^	1201.40 ± 82.27^b^	63.47 ± 4.65^ab^	1055.93 ± 112.00^ab^	54.17 ± 2.62^a^	901.20 ± 81.85^ab^	59.98 ± 2.09^a^	1074.53 ± 37.36^b^
9	HFD 8	66.79 ± 6.93^a^	1196.63 ± 124.20^b^	60.93 ± 2.92^ab^	1013.66 ± 91.80^ab^	61.73 ± 2.62^a^	1026.95 ± 90.16^b^	57.14 ± 2.35^a^	1023.72 ± 42.01^b^

*Note:* Groups with similar alphabets = no significant difference (*p* > 0.05). Values are expressed as mean ± SD, *n* = 6.

Abbreviations: HFD 1–8, high‐fat diet 1–8; NC, normal control.

However, energy intake in groups fed high‐fat diet was higher (*p* < 0.05) than the normal control at 4 weeks (Table 6), indicating clearly that the HFD was calorie dense. It is striking to note that whereas there was no observable difference in feed intake (quantity of food consumed) across groups, the overall calorie intake of the HFD‐fed rats was significantly higher than the ND‐fed rats (*p* < 0.05).

### 3.2 Confirmation of Obesity Induction

Table 7 presents the changes in body weight and related anthropometric measurements after 4 weeks of high‐fat diet feeding used to induce obesity. The results showed that rats fed the high‐fat diet had a marked increase in body weight gain compared with the normal control group. By the fourth week, percentage body weight gain in the high‐fat diet groups was significantly higher than the normal control, ranging from 24.5% to 48.8% (*p* < 0.05), confirming successful obesity induction.

**TABLE 7:** Body weight (g) changes during induction of obesity (Week 0 to Week 4).

Group	Week 0	Week 4	0–4	% weight gain compared with normal control
NC	152.25 ± 7.27^ab^	190.30 ± 10.42^b^	38.06 ± 5.38^b^	
HFD 1	156.69 ± 4.18^ab^	231.05 ± 18.17^a^	74.36 ± 18.41^a^	48.8
HFD 2	168.84 ± 8.58^a^	219.22 ± 13.26^a^	50.39 ± 11.09^ab^	24.50
HFD 3	151.62 ± 5.96^ab^	209.11 ± 6.15^a^	57.49 ± 3.80^ab^	33.80
HFD 4	159.49 ± 11.23^ab^	210.52 ± 14.65^a^	51.03 ± 5.13^ab^	25.42
HFD 5	151.04 ± 5.40^ab^	207.66 ± 5.08^a^	56.62 ± 5.59^ab^	32.80
HFD 6	141.16 ± 4.27^b^	209.15 ± 6.61^a^	67.99 ± 9.68^a^	44.00
HFD 7	161.40 ± 9.75^ab^	213.36 ± 13.07^a^	51.96 ± 13.13^ab^	26.80
HFD 8	164.41 ± 6.20^ab^	225.50 ± 15.61^a^	61.09 ± 13.77^a^	37.70

*Note:* Groups with similar alphabets = no significant difference (*p* > 0.05). Values are expressed as mean ± SD, *n* = 6.

Abbreviations: HFD 1–8, high‐fat diet 1–8; NC, normal control.

Other obesity‐related indices followed a similar pattern. Abdominal circumference, Lee's index, and body mass index were all significantly elevated in the high‐fat diet‐fed groups compared with the normal control (*p* < 0.05). Lee's index values above 310 in the high‐fat diet groups further supported the establishment of obesity. The corresponding ranges for abdominal circumference, Lee's index, and BMI were 15.50 ± 0.10–17.33 ± 0.18 vs. 14.70 ± 0.10, 362.22 ± 4.10–410.77 ± 9.16 vs. 297.25 ± 4.44, and 0.80 ± 0.06–1.04 ± 0.06 vs. 0.58 ± 0.03, respectively (Table 8).

**TABLE 8:** Anthropometric indices after obesity induction (at week 4).

Groups	Treatment	BMI (Pre‐treatment)	Lees Index (Pre‐treatment)	Abdominal circumference (Pre‐treatment)
1	NC	0.58 ± 0.03^a^	297.25 ± 4.41^a^	14.70 ± 0.10^a^
2	HFD 1	0.94 ± 0.11^bc^	363.79 ± 2.11^bc^	17.33 ± 0.18^e^
3	HFD 2	0.88 ± 0.07^bc^	375.02 ± 9.47^bcd^	16.50 ± 0.16^cd^
4	HFD 3	0.84 ± 0.06^b^	366.22 ± 9.35^bcd^	17.00 ± 0.20^de^
5	HFD 4	1.04 ± 0.06^c^	410.77 ± 9.16^f^	15.50 ± 0.10^b^
6	HFD 5	0.80 ± 0.06^b^	362.87 ± 8.67^b^	16.50 ± 0.11^cd^
7	HFD 6	0.82 ± 0.02^b^	367.24 ± 4.28^bc^	16.10 ± 0.20^c^
8	HFD 7	0.93 ± 0.05^bc^	388.48 ± 7.63^def^	16.50 ± 0.20^cd^
9	HFD 8	0.97 ± 0.06^bc^	393.07 ± 7.74^ef^	17.25 ± 0.14^e^

*Note:* Groups with similar alphabets = no significant difference (*p* > 0.05). Values are expressed as mean ± SD, *n* = 6.

Abbreviations: BMI, Body mass index; HFD 1–8, high‐fat diet groups 1–8; NC, normal control.

### 3.3 Effect of the Nut Extract

**T**he animals in this phase were obese, except the normal control. As noted earlier, intervention with *Coula edulis* extract at a two‐dose level was done in two batches. In batch 1, high‐fat diet was maintained alongside the extract administration, whereas in batch 2, the high‐fat diet was replaced with normal diet during extract administration phase. Quite fascinating results were obtained:

#### 3.3.1 Effect of Extract on Diet Intake

Diet consumption pattern within the 5 weeks of extract administration was not significantly different, comparing the HFD‐fed and the ND‐fed group (Table 9). Despite the no significant difference in quantity of diet consumed across the study groups, the overall calorie intake was found to be higher in groups fed HFD alongside extract administration, compared both to normal control and groups where HFD was replaced with ND (*p* < 0.05). Replacement of the HFD with ND within 5‐week extract administration period significantly decreased calorie/energy intake (*p* < 0.05), as can be seen in energy intake of groups 6–9 (Table 9).

**TABLE 9:** Mean daily diet and energy intake during treatment (week 5 to week 9).

Groups	Treatment	Week 5		Week 6		Week 7		Week 8		Week 9	
		Diet intake (g)	Energy intake (KJ)	Diet intake (g)	Energy intake (KJ)	Diet intake (g)	Energy intake (KJ)	Diet intake (g)	Energy intake (KJ)	Diet intake (g)	Energy intake (KJ)
1	NC	53.71 ± 6.54^a^	586.84 ± 97.22^a^	55.33 ± 3.96^a^	654.96 ± 68.71^ab^	57.33 ± 3.18^a^	678.64 ± 64.31^a^	55.24 ± 4.03^a^	653.90 ± 69.21^a^	62.86 ± 3.96^ab^	629.59 ± 99.29^a^
2	HFD + NT	58.88 ± 4.58^a^	904.15 ± 89.94^b^	57.35 ± 5.05^a^	954.13 ± 111.43^cd^	60.19 ± 3.86^a^	1001.31 ± 100.19^b^	58.83 ± 3.94^a^	978.74 ± 99.66^bc^	60.98 ± 5.73^ab^	858.40 ± 147.74^a^
3	HFD + OR	55.12 ± 4.88^a^	846.44 ± 101.39^b^	57.32 ± 5.54^a^	920.53 ± 96.57^cd^	64.18 ± 2.84^a^	1067.69 ± 94.68^b^	52.44 ± 6.06^a^	872.47 ± 120.93^abc^	69.74 ± 3.82^b^	981.75 ± 151.82^b^
4	HFD + CE1g	56.63 ± 4.15^a^	869.65 ± 95.10^b^	50.31 ± 3.76^a^	836.91 ± 89.60^cd^	61.05 ± 3.06^a^	1015.70 ± 93.19^b^	61.45 ± 2.80^a^	1022.24 ± 91.36^c^	65.23 ± 3.46^ab^	918.26 ± 141.45^b^
5	HFD + CE2g	56.72 ± 5.48^a^	871.00 ± 92.33^b^	60.46 ± 3.44^a^	1005.88 ± 96.17^d^	55.06 ± 3.71^a^	916.02 ± 93.50^ab^	62.52 ± 3.72^a^	1040.17 ± 101.07^c^	51.69 ± 2.52^a^	727.68 ± 111.07^b^
6	ND + NT	53.72 ± 2.65^a^	587.00 ± 72.41^a^	54.15 ± 4.25^a^	640.93 ± 70.31^a^	63.02 ± 4.94^a^	745.97 ± 81.83^a^	62.60 ± 4.93^a^	740.95 ± 81.47^ab^	61.24 ± 4.30^ab^	613.37 ± 98.55^a^
7	ND + OR	57.27 ± 4.53^a^	625.74 ± 86.23^a^	52.92 ± 5.80^a^	626.42 ± 83.73^a^	56.31 ± 5.79^a^	666.56 ± 85.47^a^	56.04 ± 6.51^a^	663.34 ± 92.27^a^	65.29 ± 5.00^ab^	704.18 ± 101.85^a^
8	ND + CE1g	54.15 ± 3.14^a^	591.64 ± 75.16^a^	58.53 ± 4.36^a^	692.76 ± 74.05^abc^	60.04 ± 4.15^a^	710.66 ± 73.39^a^	54.79 ± 5.80^a^	648.50 ± 84.67^a^	64.48 ± 4.96^ab^	645.83 ± 105.67^a^
9	ND + CE1g	57.65 ± 3.53^a^	629.87 ± 80.94^a^	60.85 ± 4.14^a^	720.31 ± 73.91^abc^	62.41 ± 3.43^a^	738.67 ± 69.76^a^	54.69 ± 5.16^a^	647.36 ± 78.65^a^	65.17 ± 4.24^ab^	702.98 ± 97.72^a^

*Note:* Groups with similar alphabets = no significant difference (*p* > 0.05). Values are expressed as mean ± SD, *n* = 6.

Abbreviations: CE1g, 1 g of *Coula edulis* extract; CE2g, 2 g of *Coula edulis* extract; HFD, high‐fat diet; NC, normal control; ND, normal diet; NT, no treatment; OR, orlistat.

#### 3.3.2 Effect of Extract on Body Weight and Anthropometric Indices

In this phase, the obese control recorded a steady and consistent increase in body weight, translating to about 60% higher than the normal control (*p* < 0.05). However, 5‐week administration of 1 g and 2 g of *Coula edulis* nut extracts reduced the body weight gain by 72.9% and 92.2% respectively compared to the obese control (*p* < 0.05) in batch 1 (Table 10). The corresponding value for the standard drug was determined to be 63.6%.

**TABLE 10:** Body weight (g) difference and % change in body weight during treatment.

Group	Body weight difference (week 9 – week 5)	% compared with normal (9–5)
NC	23.07 ± 2.67^b^	
HFD + NT	58.35 ± 7.42^a^	60.46
HFD + OR	21.24 ± 10.94^b^	−8.65
HFD + CE1g	15.80 ± 4.27^b^	−46.07
HFD + CE2g	4.53 ± 1.27^c^	−409.89
ND + NT	13.20 ± 7.94^b^	−74.82
ND + OR	9.48 ± 2.40^b^	−143.45
ND + CE1g	4.92 ± 2.04^cd^	−368.64
ND + CE2g	3.21 ± 8.19^b^	−378.04

*Note:* Groups with similar alphabets = no significant difference (*p* > 0.05). Values are expressed as mean ± SD, *n* = 6.

Abbreviations: CE1g, 1 g of *Coula edulis* extract; CE2g, 2 g of *Coula edulis* extract; HFD, high‐fat diet; NC, normal control; ND, normal diet; NT, no treatment; OR, orlistat.

Discontinuation of HFD feeding (ND + NT) in batch 2 caused a significant reduction in body weight gain (77.4%) relative to high‐fat diet fed rats (HFD + NT), implying that mere withdrawal of the high‐fat diet in obese patients can cause a profound positive change. With intervention, the 5‐week administration of 1 g/kg and 2 g/kg *Coula edulis* nut extract (ND + CE1g and ND + CE2g) was found to cause a further reduction in body weight gain by 62.7% and 75.7% respectively compared to the obese control in batch 2 (ND + NT) (*p* < 0.05), indicating that the effect of the high‐fat diet withdrawal was significantly complemented by extract administration (Table 10). Correspondingly, 68.8% and 29.1% significant decreases were also observed compared to the obese control in batch 1 (HFD + NT).

Administration of 2‐dose level of *Coula edulis* extract elicited interesting changes in anthropometric parameters. Extract (HFD + CE1g and HFD + CE2g) administration significantly reduced abdominal circumference, Lee's index, and BMI by (12.4%, 19.32%, 47.7%) and (17.4%, 14%, 45%) respectively compared with obese control in batch 1 (HFD + NT) (Table 12). Moreover, administration of standard drug decreased abdominal circumference, Lee's index, and BMI by 12.4%, 21.1%, 50.5% respectively compared with obese control in batch 1 (HFD + NT) (Table 12). Also, it was observed that abdominal circumference, Lee's index, and BMI of obese control in batch 2 (ND + NT) reduced significantly by 16.4%, 18.3%, and 47.7% respectively compared with HFD fed obese control (HFD + NT). Upon administration of 1 g/kg and 2 g/kg of the extract in batch 2, there was further reduction (*p* < 0.05) in BMI (17.5% and 14%) and Lee's index (5.5% and 2.7%). However, no significant difference was observed in the abdominal circumference. These observed effects were however, not dose‐dependent (Table 11).

**TABLE 11:** Anthropometric indices post *Coula edulis* treatment.

Groups	Treatment	BMI (Post‐treatment)	Lees Index (Post‐treatment)	Abdominal circumference (Post‐treatment)
1	NC	0.57 ± 0.03^a^	297.25 ± 5.65^abc^	19.00 ± 0.55^ab^
2	HFD + NT	1.09 ± 0.20^b^	378.28 ± 3.46^d^	22.25 ± 0.24^c^
3	HFD + OR	0.54 ± 0.04^a^	298.35 ± 7.33^ab^	19.50 ± 0.10^b^
4	HFD + CE1g	0.57 ± 0.02^a^	305.18 ± 5.12^abc^	19.50 ± 0.16^b^
5	HFD + CE2g	0.60 ± 0.01^a^	325.20 ± 3.90^c^	18.38 ± 0.13^a^
6	ND + NT	0.57 ± 0.03^a^	308.90 ± 3.94^bc^	18.60 ± 0.28^ab^
7	ND + OR	0.51 ± 0.02^a^	293.72 ± 4.16^a^	19.25 ± 0.11^ab^
8	ND + CE1g	0.47 ± 0.02^a^	291.15 ± 6.62^a^	18.75 ± 0.14^ab^
9	ND + CE2g	0.49 ± 0.02^a^	300.42 ± 2.80^a^	19.00 ± 0.10^ab^

*Note:* Groups with similar alphabets = no significant difference (*p* > 0.05). Values are expressed as mean ± SD, *n* = 6.

Abbreviations: BMI, Body mass index; CE1g, 1 g of *Coula edulis* extract; CE2g, 2 g of *Coula edulis* extract; HFD, high‐fat diet; NC, normal control; ND, normal diet; NT, no treatment; OR, orlistat.

### 3.4 Effect of Extract on Serum Lipid Profile and Pancreatic Lipase Activity

**FIGURE 1:**
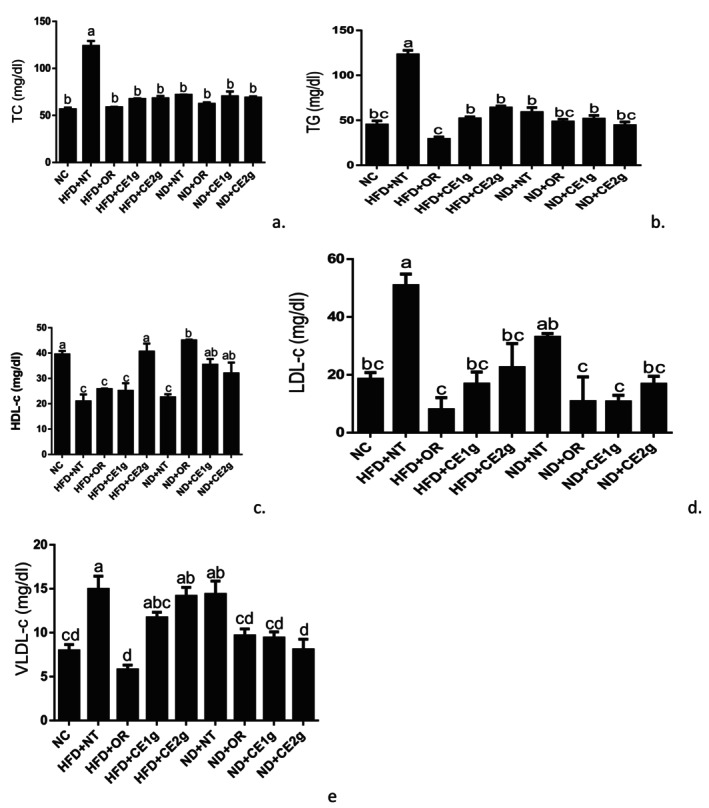
(a–e) Serum lipid profile of *Coula edulis* nuts extract treated obese rats. CE, *Coula edulis*; HDL‐c,high density lipoprotein cholesterol; HFD, high‐fat diet; LDL‐c, low density lipoprotein cholesterol; NC, normal control; ND, normal diet; NT, no treatment; OR, Orlistat; TC, total cholesterol; TG, triacylglycerol; VLDL‐c, very low density lipoprotein cholesterol. Groups with similar alphabets = no significant difference (*p* > 0.05). Values are expressed as the mean ± SD, *n* = 6.

**FIGURE 2:**
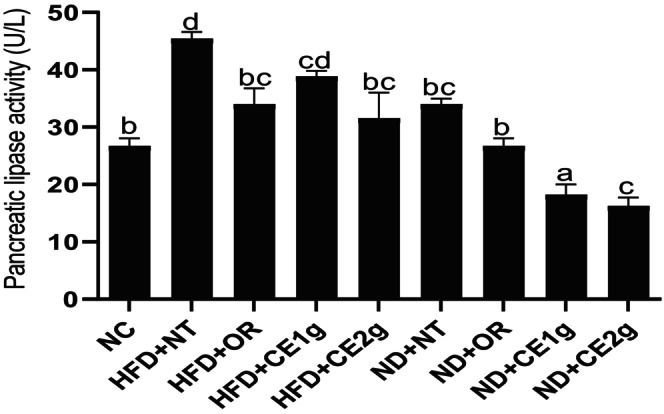
Pancreatic lipase activity of *Coula edulis* nuts extract treated obese rats. CE, *Coula edulis*; HFD, high‐fat diet; NC, normal control; ND, normal diet; NT, no treatment; OR, Orlistat. Groups with similar alphabets = no significant difference (*p* > 0.05). Values are expressed as the mean ± SD, *n = 6*.

As Figure 1 shows, the extract of Coula edulis nuts was found to have an effect on the serum lipid profile, Table 12 on cardiovascular functions indices and Figure 2 on pancreatic lipase activity. These findings showed that obese control cohorts of rats had high levels of serum total cholesterol (TC), triacylglycerol (TAG), low‐density lipoprotein cholesterol (LDL‐c) and very‐low‐density lipoprotein cholesterol (VLDL‐c) and reduced amounts of high‐density lipoprotein cholesterol (HDL‐c) as compared to normal control cohorts of rats, and thus supported the establishment of hyperlipidaemia in obesity. However, 5‐week daily administration of *Coula edulis* extract (1 g/kg and 2 g/kg) caused reduction (*p* < 0.05) in serum TC (45.6% and 48.1%), TAG (57.9% and 48.1%), LDL‐c (67% and 55.8%) and VLDL‐c (21.8% and 5.4%) and increased (*p* < 0.05) HDL‐c (18.9% and 48.4%) compared to HFD + NT. Similarly, Orlistat, a positive control in this study decreased TAG, TC, LDL‐c, VLDL‐c and increased HDL‐c levels (*p <* 0.05) by 76.5%, 45.6%, 84.4%, 61.3% and 18.9% respectively compared with the obese control in batch 1. Moreover, a 5‐week replacement of high‐fat diet with normal diet (i.e., ND + NT) alone, was seen to modulate serum lipid profile positively—reduction in TC (42%), TAG (52.3%), LDL‐c (35.1%) and a significant increase in HDL‐c (7.1%) relative to HFD + NT (*p* < 0.05). Further administration of *Coula edulis* nut extract (1 g/kg and 2 g/kg) to this batch 2 animals, sustained the positive effect on the serum lipid profile (Figure 1a–e). Again, the effect on serum lipid profile in both batches were not dose‐dependent.

**TABLE 12:** Atherogenic coefficient, atherogenic index, and cardiac risk ratio of the obese rats post *Coula edulis* nut extract treatment.

Groups	Treatment	Atherogenic coefficient (AC)	Atherogenic index (AI)	Cardiac risk ratio (CRR)
1	NC	0.18 ± 0.09^a^	0.10 ± 0.01^ab^	1.00 ± 0.04^a^
2	HFD + NT	0.72 ± 0.04^e^	0.23 ± 0.01^d^	1.72 ± 0.04^d^
3	HFD + OR	0.39 ± 0.05^abc^	0.14 ± 0.02^bc^	1.39 ± 0.05^bc^
4	HFD + CE1g	0.39 ± 0.03^abc^	0.14 ± 0.01^bc^	1.39 ± 0.03^bc^
5	HFD + CE2g	0.26 ± 0.12^ab^	0.07 ± 0.05^a^	1.26 ± 0.12^b^
6	ND + NT	0.28 ± 0.03^ab^	0.11 ± 0.01^ab^	1.24 ± 0.01^b^
7	ND + OR	0.21 ± 0.03^bcd^	0.15 ± 0.01^bc^	1.11 ± 0.03^a^
8	ND + CE1g	0.20 ± 0.05^cde^	0.11 ± 0.01^abc^	1.19 ± 0.05^b^
9	ND + CE2g	0.18 ± 0.07^cd^	0.10 ± 0.02^abc^	1.16 ± 0.07^bcd^

*Note:* Groups with similar alphabets = no significant difference (*p* > 0.05). Values are expressed as the mean ± SD, *n* = 6. AC = (TC‐HDL‐c)/HDL‐c, CRR = TC/HDL‐c, AI = log (TG/HDL‐c).

Abbreviations: CE, *Coula edulis*; HFD, high‐fat diet; NC, normal control; ND, normal diet; NT, no treatment; OR, Orlistat.

As depicted in Table 12, atherogenic index, atherogenic coefficient and cardiac risk ratio in the high‐fat diet obese control increased by 56.5%, 75% and 41.9% respectively compared with the normal control (*p* < 0.05). But following a 5‐week intervention with *Coula edulis* nut extract (1 g/kg and 2 g/kg) modulated these derived indices thus: atherogenic index, atherogenic coefficient and cardiac risk ratio by 39.1% and 69.6%, 45.8% and 63.9%, and 19.2% and 26.7% respectively compared to HFD + NT (*p* < 0.05). This decrease occurred in a dose‐dependent manner. Interestingly, the atherogenic index and atherogenic coefficient of rats in the extract treated groups were not significantly different from that of normal control suggesting a complete reversal/normalization of the obesity‐induced cardiovascular risk. In the second batch, it was found that replacement of HFD with ND alone positively attenuated the cardiovascular risk indicators (*p* < 0.05). Administration of the nut extract (1 g/kg and 2 g/kg) in this batch further reduced the cardiovascular risk (*p* < 0.05). Precisely, administration at 2 g/kg decreased atherogenic coefficient by 30.8% compared with 2 g/kg of the extract in batch 1 (*p* < 0.05).

Serum pancreatic lipase activity was also evaluated in order to assess the impact of the nut on dietary triacylglycerol digestion/hydrolysis. High‐fat diet control showed a remarkable increase (*p* < 0.05) in serum lipase activity by 41.2% as compared to the normal control (Figure 2) indicating an active hydrolysis of fats. However, a 5‐week administration of *Coula edulis* nut extract (1 g/kg and 2 g/kg) was found to cause a decrease in the activity of the enzyme by 14.5% and 30.5% as compared to HFD + NT (*p* < 0.05) in a dose dependent manner. Substitution of the high fat diet with normal diet as shown in batch 2 was seen to attenuate the serum pancreatic lipase activity by 25.1% (i.e., HFD + NT vs. ND + NT). Treatment with the reference drug, 1 g/kg and 2 g/kg of *Coula edulis* further reduced (*p* < 0.05) serum pancreatic lipase activity by 27.3%, 46.4% and 52.2% respectively compared with ND + NT. Serum pancreatic lipase activity in ND + CE2g decreased significantly compared with HFD + CE2g by 48.5%.

### 3.5 Effect of Extract on Tissue Lipids, Weights and Hepatic HMG‐CoA Reductase Activity

**FIGURE 3:**
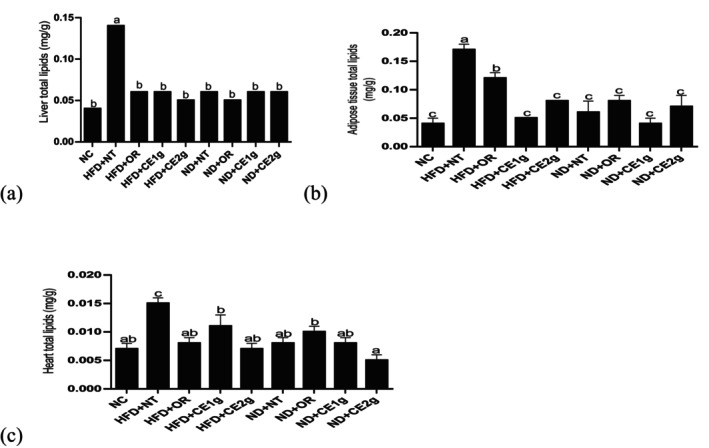
(a–c) Effect of *Coula edulis* nut extract on total lipids (mg/g) of liver, adipose, and heart of obese rats. CE, *Coula edulis*; HFD, high‐fat diet; NC, normal control; ND, normal diet; NT, no treatment; OR, Orlistat. Groups with similar alphabets = no significant difference (*p* > 0.05). Values are expressed as the mean ± SD, *n = 6*.

The impact of Coula edulis extract on the total lipids of tissues/organs is illustrated in Figure 3a–c. The total lipid levels in the heart, liver, and adipose tissue of the HFD + NT (obese control) group increased by 53.3%, 71.4%, and 76.5%, respectively, in comparison to the normal control (*p* < 0.05). Nonetheless, a 5‐week dose of Coula edulis nut extract (1 g/kg and 2 g/kg) markedly diminished total lipids in the heart, liver, and adipose tissue by 26.7% and 53.3%, 57.1% and 64.2%, and 70.5% and 52.9%, respectively, compared to the obese control (*p* < 0.05). The observed effect was dose‐dependent, with the exception of adipose tissue. The extract's effect was comparable to that of the usual medication. The removal of the HFD in batch 2 alone mitigated the adverse effects of HFD on total lipids in tissues and organs. Total lipids in the heart, liver, and adipose tissue reduced considerably by 46.7%, 57.1%, and 64.7%, respectively, compared to the high‐fat diet obese control (*p* < 0.05). The administration of the extract in this group of animals (batch 2) revealed no significant difference in total lipid levels.

**FIGURE 4:**
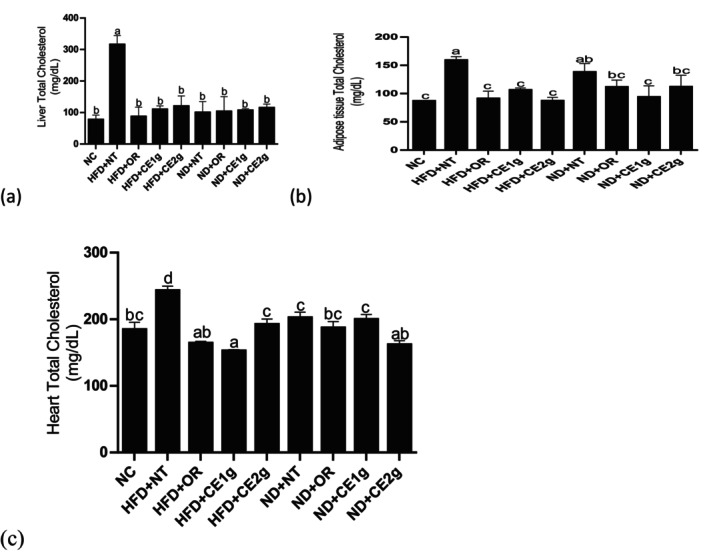
(a–c) Effect of *Coula edulis* nut extract on total cholesterol concentration (mg/dL) of liver, adipose tissue and heart of obese rats. CE, *Coula edulis*; HFD, high‐fat diet; NC, normal control; ND, normal diet; NT, no treatment; OR, Orlistat. Groups with similar alphabets = no significant difference (*p* > 0.05). Values are expressed as the mean ± SD, *n = 6*.

**FIGURE 5:**
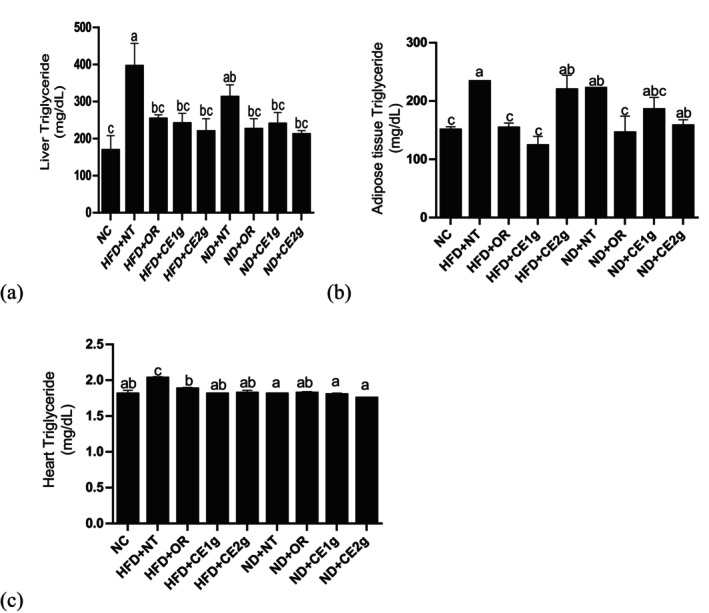
(a–c) Effect of *Coula edulis* nut extract on triacylglycerol concentration (mg/dL) of liver, adipose tissue and heart of obese rats. CE, *Coula edulis*; HFD, high‐fat diet; NC, normal control; ND, normal diet; NT, no treatment; OR, Orlistat. Groups with similar alphabets = no significant difference (*p* > 0.05). Values are expressed as the mean ± SD, *n = 6*.

The total cholesterol level was assessed in the heart, adipose tissue, and liver. The data acquired is illustrated in Figure 4a–c. Total cholesterol levels in the heart, liver, and adipose tissue markedly elevated in the obese control rats (HFD + NT) by 24%, 75.6%, and 45.5%, respectively, in comparison to the normal control group. Upon administration of the extract at doses of 1 g/kg and 2 g/kg, total cholesterol levels diminished in the heart (37.2% and 20.8%), liver (65.2% and 61.9%), and adipose tissue (33.3% and 45.2%) relative to the high‐fat diet obese control (*p* < 0.05). The cessation of a high‐fat diet and its substitution with a normal diet in batch 2 (ND + NT) mitigated the impact of obesity on total cholesterol levels in the heart (16.7%), liver (68.2%), and adipose tissue (13.2%) when compared to the high‐fat diet‐fed obese control (*p* < 0.05). Treatment with the extract (1 g/kg and 2 g/kg) significantly diminished the accumulated cholesterol in adipose tissue by 32.1% and 19%, respectively (*p* < 0.05). The extract resulted in a substantial 20% reduction in cardiac total cholesterol at a dosage of 2 g/kg (*p* < 0.05), showing an effect with greater intake. Tissue triacylglycerol reserves were significantly higher (*p* < 0.05) in obese control rats (HFD + NT) throughout all examined tissues compared to rats on a normal diet (Figure 5a–c). In the heart, triacylglycerol levels rose by 10.8% (2.03 ± 0.02 vs. 1.81 ± 0.05), in the liver by 57.4% (395.60 ± 61.48 vs. 168.46 ± 39.86), and in adipose tissue by 35.6% (233.58 ± 0.01 vs. 150.36 ± 0.01). After a 5‐week oral treatment of Coula edulis nut extract (1 g and 2 g), triacylglycerol levels reduced by 10.8% and 10.3%, 39% and 44.6%, and 47.1% and 6.5% in the heart, liver, and adipose tissue, respectively (*p* < 0.05). In batch 2, where high‐fat diet (HFD) was substituted with normal diet (ND) throughout a 5‐week duration, a significant reduction (*p* < 0.05) in triacylglycerol levels was noted: a decrease of 10.8% in the heart, 21.1% in the liver, and 5% in adipose tissue (HFD + NT vs. ND + NT). The administration of the extract (1 g/kg and 2 g/kg) led to a further decrease in triacylglycerol levels in the heart (0.5% and 3.5%), liver (23.2% and 32.2%), and adipose tissue (15.6% and 28.9%), albeit without statistical significance.

**FIGURE 6:**
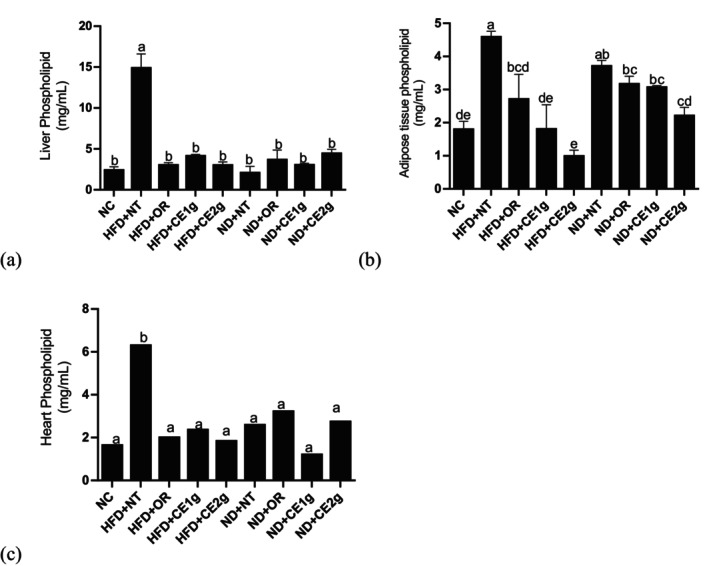
(a–c) Effect of *Coula edulis* nut extract on phospholipid concentration (mg/mL) of liver, adipose tissue, and heart of obese rats. CE, *Coula edulis*; HFD, high‐fat diet; NC, normal control; ND, normal diet; NT, no treatment; OR, Orlistat. Groups with similar alphabets = no significant difference (*p* > 0.05). Values are expressed as the mean ± SD, *n = 6*.

The effect of extract administration on tissue phospholipid is illustrated in Figure 6a–c. The accumulation of phospholipids significantly increased (*p* < 0.05) in the obese control group (HFD + NT) compared to the normal control, with a rise of 73.8% in the heart (6.29 ± 1.75 vs. 1.65 ± 0.45), 84% in the liver (14.86 ± 1.75 vs. 2.38 ± 0.42), and 60.9% in adipose tissue (4.58 ± 0.18 vs. 1.79 ± 0.25). Following intervention with 1 g/kg and 2 g/kg of the extract, phospholipid levels increased (*p* < 0.05) by 62.6% and 71% in the heart, 72.3% and 81.4% in the liver, and 60.7% and 78.6% in adipose tissue, relative to the obese control (HFD + NT), in a dose‐dependent manner. Withdrawal from a high‐fat diet during the intervention period significantly enhanced phospholipid reserves in the heart and liver by 59% and 86.2% (*p* < 0.05). Nevertheless, the enhancement in the adipose tissue was not statistically significant. The dosing in this batch did not induce substantial alterations in phospholipids compared to the control (ND + NT).

**FIGURE 7:**
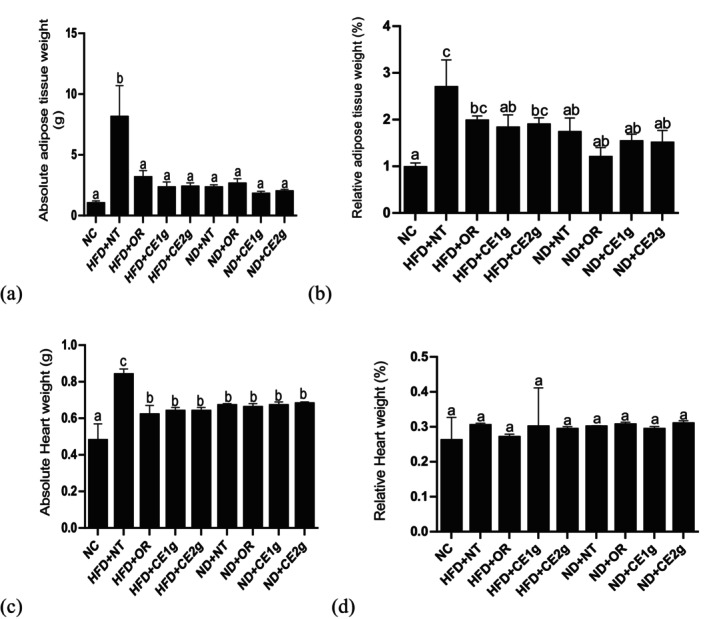
(a–d) Effect of *Coula edulis* nut extract on absolute (g) and relative weight (%) of adipose tissue and heart in high‐fat diet‐induced obese rats. CE, *Coula edulis*; HFD, high‐fat diet; NC, normal control; ND, normal diet; NT, no treatment; OR, Orlistat. Groups with similar alphabets = no significant difference (*p* > 0.05). Values are expressed as the mean ± SD, *n* = 6.

Figure 7a–d illustrates the impact of the intervention on both absolute and relative weights of adipose tissue and the heart. The absolute and relative weights of the obese control (HFD + NT) were elevated in the adipose tissue (87.6% and 63.6%) and heart (42.9% and 13.33%) compared to the normal control. All effects are statistically significant, with the exception of the relative heart weight. Treatment with nut extract (1 g/kg and 2 g/kg) significantly diminished (*p* < 0.05) the absolute weight of adipose tissue by 71.6% and 70.8%, the relative weight of adipose tissue by 32.2% and 30%, and the absolute weight of the heart by 23.4% and 23.41% in comparison to the high‐fat diet‐fed obese control (HFD + NT). No statistically significant difference was seen in the relative weight of the heart. The substitution of a high‐fat diet with a standard diet significantly influenced both the absolute and relative weights of adipose tissue, as well as the absolute weight of the heart: it decreased the absolute and relative weights of adipose tissue by 71.7% and 35.8%, respectively, and reduced the absolute heart weight by 20.2% in comparison to the high‐fat fed obese control (HFD + NT) group (*p* < 0.05). The treatment further decreased the weights, albeit not to a statistically significant degree. The results indicate that the extract's effect was more significant than that of the reference medicine, Orlistat.

**FIGURE 8:**
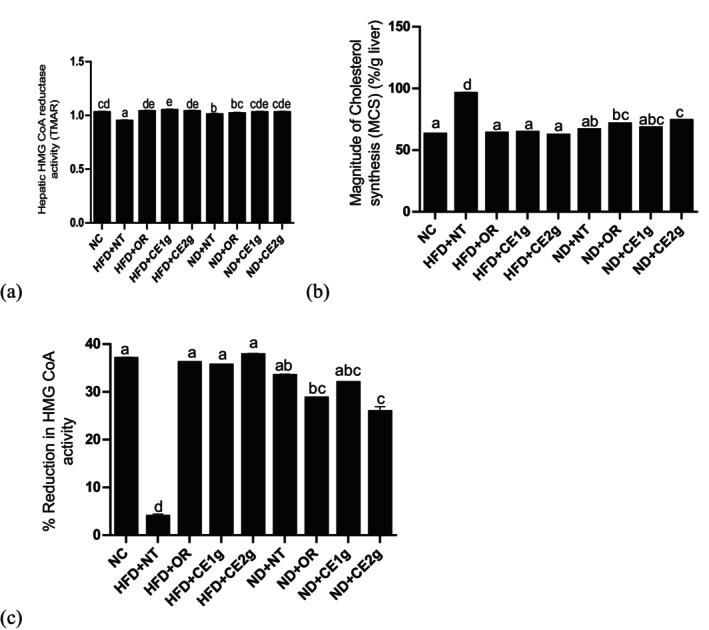
(a–c) Effect of *Coula edulis* nut extract on hepatic HMG CoA reductase activity (TMAR), magnitude of cholesterol synthesis (MCS) in the liver, and % reduction in HMG CoA reductase activity of obese rats. CE, *Coula edulis*; HFD, high‐fat diet; MCS, magnitude of cholesterol synthesis; NC, normal control; ND, normal diet; NT, no treatment; OR, Orlistat; TMAR, tissue mevalonic acid ratio. Groups with similar alphabets = no significant difference (*p* > 0.05). Values are expressed as the mean ± SD, *n = 6*.

The impact of this medication on the hepatic HMG‐CoA/Mevalonic acid ratio (TMAR), an indication of HMG‐CoA reductase activity, is illustrated in Figure 8a. The TMAR was reduced by 7.8% (*p* < 0.05) in the high‐fat diet obese control compared to rats on a normal diet, indicating elevated HMG‐CoA reductase activity in the obese control, signifying enhanced cholesterol production in obesity. After administration of Orlistat and Coula edulis nut extract (1 g/kg and 2 g/kg), TMAR increased by 8.65%, 9.52%, and 8.65%, respectively, compared to the untreated obese rats, indicating diminished HMG‐CoA reductase activity in the treatment groups, thereby suggesting a reduction in hepatic cholesterol biosynthesis. The extract's effect was comparable to that of the regular medication. The cessation of the high‐fat diet during the intervention period resulted in a 5.94% rise in TMAR (*p* < 0.05) compared to untreated obese rats that continued the high‐fat diet (i.e., HFD + NT versus ND + NT), indicating that the withdrawal of HFD suppresses cholesterol production in the liver. An additional rise of TMAR by 1.94% (*p* < 0.05) was noted upon administration of the extract in two doses.

Table 13 illustrates the association between TMAR and hepatic cholesterol levels. A robust negative Pearson correlation coefficient (−0.637) was noted. This indicates that as TMAR values rise, the total cholesterol concentration in the liver diminishes, which therefore results in a reduction of HMG‐CoA reductase activity. It originates from mevalonate, the denominator in the TMAR ratio, serving as the precursor for cholesterol production.

**TABLE 13:** Correlation between HMG‐CoA/Mevalonic acid ratio and hepatic cholesterol concentration.

Groups	Hepatic total cholesterol (mg/dL)	TMAR	Pearson correlation coefficient (*r*)
NC	77.16 ± 14.71^b^	1.03 ± 0.00^cd^	
HFD + NT	315.74 ± 28.48^a^	0.95 ± 0.01^a^	
HFD + OR	87.24 ± 29.60^b^	1.04 ± 0.00^de^	
HFD + CE1g	109.88 ± 11.43^b^	1.05 ± 0.01^e^	
HFD + CE2g	120.17 ± 32.64^b^	1.04 ± 0.01^de^	
ND + NT	100.31 ± 34.63^b^	1.01 ± 0.00^b^	
ND + OR	103.09 ± 47.53^b^	1.02 ± 0.01^bc^	
ND + CE1g	106.79 ± 5.30^b^	1.03 ± 0.01^cde^	
ND + CE2g	114.82 ± 12.06^b^	1.03 ± 0.01^cde^	−0.637
			*p* = 0.05

*Note:* Groups with similar alphabets = no significant difference (*p* > 0.05). Values are expressed as the mean ± SD, *n* = 6.

Abbreviations: CE, *Coula edulis*; HFD, high‐fat diet; HMG‐CoA, hydroxymethylglutaryl coenzyme A; NC, normal control; ND, normal diet; NT, no treatment; OR, Orlistat; TMAR, Tissue Mevalonic acid ratio.

Figure 8b illustrates the impact of the medication on the extent of cholesterol production in the liver, quantified as the percentage of mevalonic acid synthesis per gram of liver weight. The extent of hepatic cholesterol production was determined to be 96.04% (*p* < 0.05) in the obese control group of batch 1 (HFD + NT), compared to 62.98% in the normal control group. Following a 5‐week treatment with 1 g/kg and 2 g/kg of the nut extract, hepatic cholesterol production decreased significantly (*p* < 0.05) to 64.38% and 62.21%, respectively, correlating with a 35.62% and 37.79% drop in HMG‐CoA reductase activity, respectively (Figure 8c). The substitution of the high‐fat diet with a standard diet during the intervention significantly diminished (*p* < 0.05) hepatic cholesterol production from 96.04% to 66.6% and enhanced the percentage reduction in HMG‐CoA reductase activity from 3.96% to 33.4%. The administration of the extract in this batch did not result in any notable alterations. This outcome reaffirms the relationship between HMG‐CoA reductase activity and cholesterol levels in the liver.

### 3.6 Effect of Extract on Oxidative Stress Indicators and Oxidant Levels in the Adipose Tissue

**FIGURE 9:**
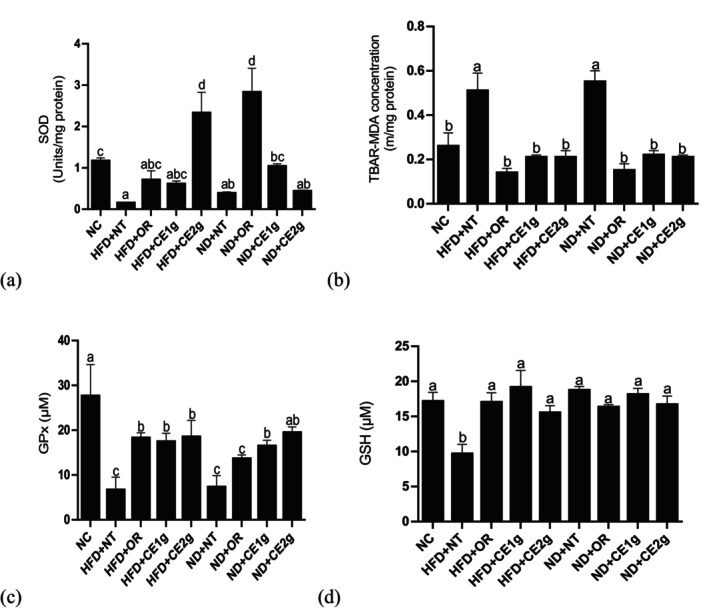
(a–d) Effect of *Coula edulis* on oxidative stress markers (SOD, GPx activity, MDA and GSH levels) in the adipose tissue of obese rats. CE, *Coula edulis*; GPx, Glutathione peroxidase; GSH, Reduced glutathione; HFD, High‐fat diet; NC, Normal control; ND, Normal diet; NT, No treatment; OR, Orlistat; SOD, Superoxide dismutase; TBARS‐MDA, Thiobarbituric acid reactive substances—Malondialdehyde. Groups with similar alphabets: No significant difference (*p* > 0.05). Values are expressed as the mean ± SD, *n = 6*.

Results of the effect of the extract on activities of SOD and GPx and levels of GSH and TBARS‐MDA concentration determined in the adipose tissue are shown in Figure 9 a‐d. From the results, the concentration of GSH, activities of SOD and GPx were depleted in the high‐fat diet obese control (HFD + NT) compared to normal control by 43.6%, 87.1% and 75.8% respectively (*p* < 0.05), indicating a stressed environment in obesity. These indicators, including SOD, GPx and GSH were significantly elevated (*p* < 0.05) following a 5‐week administration of the extract at doses 1 g/kg and 2 g/kg—(75.4% and 93.6%), (10.3% and 63.9%), and (49.5% and 37.7%) respectively relatively to the obese control (HFD + NT). This observed result showed similar trend with standard drug Orlistat. Also, suspension of the high‐fat diet in batch 2 was seen to improve the activity of SOD, GPx and GSH levels by 61.5%, 8.7%, and 48.4% respectively (i.e., ND + NT vs. HFD + NT). Administration of the nut extract (1 g/kg and 2 g/kg) to this batch, further improved the activity of SOD and GPx by (62.5% and 72.9%) and (55.6% and 62.4%) (*p* < 0.05). GSH levels did not significantly change on administration of the extract.

High‐fat diet‐induced obesity also elicited lipid peroxidation, depicted by increased TBARS‐MDA levels by 49.0% compared to normal control (*p* < 0.05). Extract administration at 1 g/kg and 2 g/kg both reduced TBARS‐MDA level by 58.8% compared with high‐fat diet obese control. Discontinuation of high fat diet in batch 2 at week 5 and replacement with normal diet had no significant impact on TBAR‐MDA when compared to the high‐fat diet obese control (ND + NT vs. HFD + NT). However, treatment with Orlistat and extract (1 g/kg and 2 g/kg) lowered lipid peroxidation by 72.7%, 60% and 61.8% respectively compared to the control (ND + NT).

### 3.7 Concentration and Purity of the Extracted RNA

The values of concentration and purity of extracted RNA of liver and adipose tissue is as shown in Table S1a,b, respectively. The concentrations of RNA extracted from the liver in the experimental groups were higher than the concentration values of RNA extracts from the adipose tissue. This may be due to higher metabolic activities in the liver compared to the adipose tissue. The purity absorbances of the RNA extracts of liver and adipose tissue were within the purity criteria (≥ 1.8 ≤ 2.2).

### 3.8 Effect of the Nut Extract on the Expression of Adipogenesis and Lipogenesis Regulatory Target (Upstream) Genes in the Adipose and Liver Tissues

**FIGURE 10:**
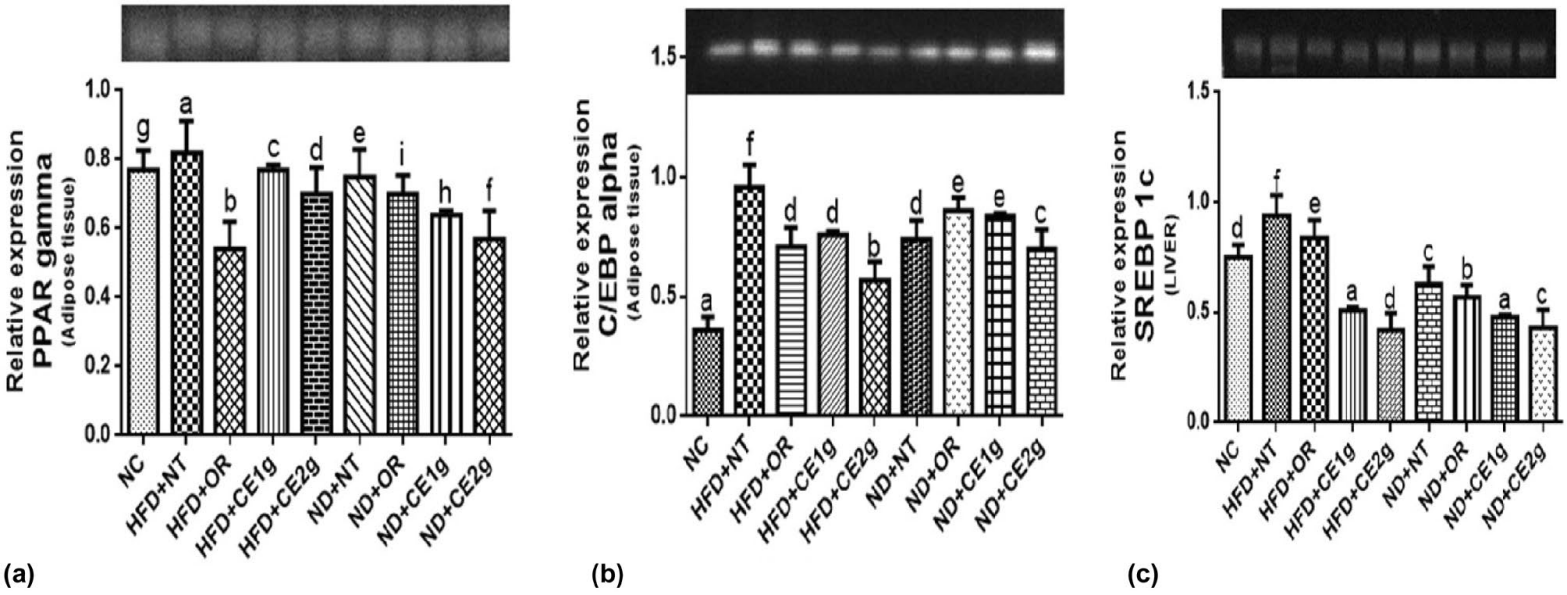
(a–c) (a) Effect of *Coula edulis* nut extract on relative expression of PPAR‐γ in adipose tissue of obese rats. Groups with similar alphabets = no significant difference (*p* > 0.05). Values are expressed as mean ± SD, *n* = 2. (b) Effect of *Coula edulis* nut extract on relative expression of C/EBP‐α in adipose tissue of obese rats. Groups with similar alphabets = no significant difference (*p* > 0.05). Values are expressed as mean ± SD, *n* = 2. (c Effect of *Coula edulis* nut extract on relative expression of SREB) P 1c in liver of obese rats. NC, normal control; HFD, high‐fat diet; NT, no treatment; OR, orlistat; CE, *Coula edulis*. Groups with similar alphabets = no significant difference (*p* > 0.05). Values are expressed as mean ± SD, *n* = 2.

The relative expressions of PPARγ, C/EBPα in the adipose tissue and SREBP‐1c in liver of obese rats treated with *Coula edulis* nut extract for 5 weeks are shown in Figure 10a–c. The result showed an up‐regulation in the expression of the transcription factor, PPARγ, C/EBPα and SREBP‐1c in obese condition (HFD + NT) by 6.1%, 62.5% and 19.9% respectively compared to normal control (*p* < 0.05). However, 5‐week treatment with 1 g/kg and 2 g/kg extract of the nut down‐regulated the expression of PPARγ by 6.0% and 14.6%, C/EBPα by 26.2% and 68.9% and SREBP‐1c by 85.9% and 25.6% relative to the high‐fat diet obese control (*p* < 0.05). Withdrawal of the high fat diet after induction of obesity, decreased the relative expression of PPARγ, C/EBPα and SREBP‐1c by 13.8%, 23.2% and 33.5% respectively compared with the high‐fat diet fed obese control (*p* < 0.05). 5‐week administration of the extract (1 g/kg and 2 g/kg) caused a further decrease in the expression of PPARγ by 9.4% and 21.7% respectively (*p* < 0.05). However, the extract was only effective at the higher dose (5.3% significant decrease in C/EBPα expression compared ND + NT), i.e., the 1 g/kg dose was not sufficient a dose to cause a response. SREBP‐1c expression was down‐regulated by 23.6% and 31.7% on administration of 1 g/kg and 2 g/kg of extract compared with ND + NT (*p* < 0.05).

### 3.9 Effect of Extract on Histology of Adipose

Plate 1‐9S showed photomicrographs of the histological changes in the adipose tissues of extract treated rats compared to the controls. H and E staining of the white adipose tissue was conducted to establish changes in the size and number of adipocytes. A summary result of the effects of the extract on adipose tissue histology of treated compared to the controls, as well as the implications of the changes are shown on Table S2.

## 4 Discussion

In this study, Wistar rats were used to study the role of high‐fat diet on metabolic health in the context of obesity. For 4 weeks, there was a significant gain in the weight of the rats that were fed a high‐fat diet as compared to the control group that was fed on a normal diet and thus confirmed the induction of obesity through dietary intervention. Notably, there were no statistically significant inter‐group differences in daily food consumption, even though the weight difference was significant, and this could be explained by the fact that the high‐calorie diet of the high‐fat regime is rich in calories. This increase in weight is plausibly attributed to an increased transformation of saturated fatty acids to triacylglycerol that can disequilibrate energy homeostasis leading to the establishment of a sustained obesogenic condition (Li et al. 2023). The high‐fat diet group exhibited considerable morphological adjustments in terms of increases in body mass index (BMI), Lee index and abdominal circumference. These changes are in line with the previous research that has shown that high‐fat intake inhibits resting metabolic rate and thermogenic response, leading to the accumulation of lipids especially in the abdominal area (Maharjan et al. 2021).

The study further tested the efficacy of *Coula edulis* nut extract at two doses, 1 g/kg and 2 g/kg body weight. Both dosages resulted in a statistically significant reduction in weight gain compared to the untreated controls, which is similar to the pharmacological effect of the anti‐obesity agent Orlistat. Rats treated with the extract continued with improvements in abdominal measures and overall weight course, thus strengthening the therapeutic potential of the extract. The weight‐regulating properties of *Coula edulis* are apparently based on its very high protein content and unsaturated fatty acids, which are constituents known to increase energy expenditure and have thermogenic properties (Naccis et al. 2025; Zhang et al. 2019). By suppressing the synthesis of lipids and promoting the oxidation of lipids by the mediation of cyclic adenosine monophosphate (cAMP) signaling pathways, the phytochemicals in the extract like phenols and flavonoids may contribute to enhanced lipolysis and metabolic rate, thus promoting weight loss (Zhang et al. 2019).

In regards to cardiovascular outcomes, it was found that cardiovascular dysfunction (dyslipidaemia) is strongly correlated with obesity and triggers other cardiovascular dysfunctions. The level of serum total cholesterol (TC), triacylglycerides, low‐density lipoprotein cholesterol (LDL‐c), and very low‐density lipoprotein cholesterol (VLDL‐c) significantly reduced, and increased high‐density lipoprotein cholesterol (HDL‐c) levels was observed compared to the untreated obese rats, that is, with the administration of *Coula edulis* extract. In most cases, obesity leads to dyslipidaemic profiles (elevated levels of triacylglycerols and reduced levels of HDL‐c) which increases the level of cardiovascular risk (Umoru et al. 2025).

Moreover, by inhibiting lipase activity, promoting hormone secretion that is linked with satiety, and reducing absorption and digestion of dietary lipids, the extract can potentially reduce lipid accumulation (Liu et al. 2020). *Coula edulis* treated obese rats showed significant improvement in lipid profile with reduced atherogenic indexes and cardiac risk ratios compared to the untreated obese controls which had higher atherogenic indexes, suggesting severe risk of cardiovascular conditions. These results comprise a treatise of the efficacy of *Coula edulis* nut extract to be considered a prospective prophylactic for obesity and its associated cardiovascular pathologies.

Our investigation showed high lipase activity in the serum of rats fed on a high‐fat diet and therefore highlighted the important role of pancreatic lipase in lipid digestion through hydrolysis of triacylglycerols into absorbable monoacylglycerols and free fatty acids. Diminished enzymatic activity may culminate in decreased fat assimilation and impaired energy introgression, factors which are necessary determinants in the administration of obesity (Banaszak et al. 2025). The study showed that an extract from the nuts of *Coula edulis* inhibits lipase activity, which is thought to be because of its phenolic and flavonoid content, which are known to inhibit the enzymes that are involved in the metabolism of fats (Zhou et al. 2023). Consequently, a decrease in lipase activity leads to a decrease in the hydrolysis of the dietary triglycerides and a decrease in serum cholesterol, triacylglycerol and low‐density lipoprotein (LDL) levels in treated rats.

Therefore, the anti‐obesity effect of the *Coula edulis* nut extract may not only be related to the total flavonoid content, but also to the combined effects of specific groups of bioactive compounds that may be present in the extract, such as flavonoids, phenolic acids, unsaturated fatty acids, phytosterols and tocopherol‐like antioxidants. Flavonoids and phenolic compounds may decrease obesity by suppressing pancreatic lipase, thus reducing the digestion of dietary triacylglycerols to absorbable free fatty acids and monoacylglycerols (Aloo et al. 2023; Mahboob et al. 2023). This is consistent with the depression in pancreatic lipase activity, triacylglycerol, low density cholesterol (LDL‐c) and lipid accumulation in tissues observed in the animals. They may also reduce adipocyte differentiation by downregulating some of the key transcription factors involved in adipocyte differentiation (PPAR‐γ and C/EBP‐α) and reducing hepatic lipogenesis through suppression of SREBP‐1c. Furthermore, unsaturated fatty acids may aid in lipid transport by enhancing fatty acid oxidation and preventing ectopic fat storage, while phytosterols may impede cholesterol absorption from the gut and contribute to decreased serum and hepatic cholesterol concentrations (Frasinariu et al. 2022). Finally, antioxidant components of the extract may also safeguard adipose tissue from the obesity‐induced oxidative stress by increasing endogenous antioxidant levels (SOD, GPx and GSH), and decreasing lipid peroxidation (Uti et al. 2020a). Overall, these mechanisms indicate that the anti‐obesity properties of Coula edulis nut extract are likely pleiotropic, involving reduction in fat digestion and absorption in the small intestine, suppression of cholesterol synthesis, inhibition of adipogenesis and lipogenesis, improvement in antioxidant defenses and reduction in fatty acids.

Furthermore, administration of the extract was correlated with a decrease in the relative weights of total fat depots, suggestive of inhibitory effects on fat cell formation and decrease in fat cell size. These results were supported by low levels of triacylglycerol, cholesterol, phospholipids, and total lipids in fat depots. The study found that the abdominal fat mass was a significant predictor of metabolic disturbances, including cardiovascular diseases, and so it is possible that the effect of the extract on body weight and fat stores might provide protection against the metabolic complications associated with obesity.

Organ weights of extract‐treated rats were lower than untreated obese controls, which correlated with reduced body weight and reduced absorption of triglycerides and cholesterol. *Coula edulis* extract was also seen to reduce hepatic triacylglycerol and cholesterol levels, parameters which are commonly associated with non‐ alcoholic fatty liver disease (NAFLD) as well as with the metabolic syndrome (Zhou et al. 2023). Hepatic steatosis, which is characterized by the over‐accumulation of lipids because of imbalances in fatty acid synthesis and catabolism, was reduced in extract treated rats (Iahtisham‐Ul‐Haq et al. 2025). This result is consistent with the finding that the extract reduced the activity of HMG‐CoA reductase to modulate cholesterol biosynthesis (Uti et al. 2020a). In addition, oxidative stress has been implicated in a variety of pathologies, such as the metabolic syndrome. Administration of *Coula edulis* extract was able to reduce lipid peroxidation as measured by concentrations of malondialdehyde (MDA), a well‐known marker of oxidative damage. In contrast, the untreated obese rats had higher MDA concentrations that suggested there may be a relationship between high dietary fat intake and high oxidative stress concentrations (Tan and Norhaizan 2019). The extract improved antioxidant defenses by the up‐regulation of antioxidant enzymes and non‐enzymatic antioxidants.

Histological examinations of adipose tissue showed that *Coula edulis* nut extract significantly decreased both the size and number of the adipocytes, presumably through modulation of key transcription factors in adipogenesis including the peroxisome proliferator‐activated receptor‐γ and its upstream transcription factor, C/EBP‐α. This modulation hindered the lipid accumulation and proliferation process of adipocytes, and this phenomenon consolidated the role of the extract to counteract obesity via a down‐regulation of genes linked to the adipogenic and lipogenic processes. Additionally, high plasma lipid levels can induce accumulation of lipids in the liver; however, administration of *Coula edulis* nut extract coincided with the reduced plasma lipid levels, suggesting a possible prophylactic effect against production of hepatic lipid accumulation (Ali et al. 2024). This observation was corroborated by a significant inhibition of the lipogenesis‐related gene expression such as hepatic SREBP‐1c, which suggests that the nut extract may be effective in modulating lipogenic pathways and inhibiting fatty acid uptake.

The suppression of PPAR‐γ, C/EBPα, and SREBP‐1c offers a mechanism for the amelioration of the distorted biochemical, anthropometric and histological parameters investigated. These two factors regulate processes of adipocyte differentiation and lipid storage, and in view of their lower expression, the lower adipose tissue weight, smaller adipocyte size, reduced BMI, Lee's index and tissue lipid content are expected. Likewise, the down‐regulation of hepatic SREBP‐1c, a key regulator of lipogenesis, is responsible for the reduction in hepatic triacylglycerol and cholesterol, improved serum lipid profile and cardiac risk factors. Therefore, genes regulate the anti‐adipogenic/anti‐lipogenic activity of the extract. In summary, the *Coula edulis* nut extract reveals quite a bit of potential in modulating the attenuation of adipogenesis and lipogenesis, as well as avoiding the accumulation of fat in the liver, as well as protective action against obesity‐associated metabolic diseases by regulating energy metabolism and oxidative stress.

## 5 Summary and Conclusion

This study investigated the anti‐obesity effects of whole *Coula edulis* nut extract and its possible molecular mechanisms in high‐fat diet‐induced obese Wistar rats. Anthropometric indices, food intake, organ and tissue weights, serum lipid profile, pancreatic lipase activity, cardiovascular risk ratios, adipose tissue lipids, ectopic fat accumulation, hepatic HMG‐CoA reductase activity, and oxidative stress markers were assessed. The extract produced beneficial effects across these parameters. It also positively modulated obesity‐related genes, including PPAR‐γ, C/EBPα, and SREBP‐1c.

Overall, the findings validate the anti‐obesity potential of *Coula edulis* nut extract, likely due to the individual, additive, or synergistic actions of its phytochemicals. The extract reduced lipid absorption, biosynthesis, storage, energy intake, oxidative damage, and inflammation, while enhancing energy expenditure and regulating pre‐adipocyte differentiation and proliferation. Its effects may be improved by reducing or stopping high‐calorie and saturated‐fat intake. In sum, the contributions of this study are summarized in Table S3a,b.

### 5.1 Study Limitations

This study has some limitations. While the high‐fat diet‐induced obesity model is the most commonly used, it does not completely simulate the complex and chronic nature of human obesity, which encompasses genetic, dietary, physical environment, lifestyle, hormone, and microbiome factors. The duration of obesity induction and treatment also restricts our ability to draw conclusions on the maintenance of weight loss, preventing weight regain and safety. Furthermore, some of the effects may have been due to high‐fat diet withdrawal itself, as obese rats given normal chow had improved metabolic indices. The high dose of ethanol extract, without isolation, quantification, and standardization of active ingredients, precludes reproducibility and dose equivalence. Lack of description regarding randomization, allocation concealment, and blinded assessment of outcomes, particularly for the histological and morphometric findings, may be sources of bias. Lastly, some outcomes such as calculated proportions of lipid fractions, indirect measurement of HMG‐CoA reductase activity, and traditional PCR analysis of gene expression could lead to unreliability. Thus, additional research is needed with standardized fractions of *Coula edulis* nut extract, direct molecular and enzyme activity parameters, detailed toxicity testing, and with longer‐term follow‐up, before its anti‐obesity effects can be reliably translated to other animals.

### 5.2 Contribution to Knowledge

1. The study has scientifically established body weight regulation activity of *Coula edulis*, i.e., established anti‐obesity activity.
2. The study nuts have been shown to effectively modulate the macrovascular changes associated with obesity.
3. The study has also demonstrated in part, the mechanisms by which the anti‐obesity function is exerted namely biochemical modulation of fat digestion, and cholesterol biosynthesis and molecular modulation of the processes of adipogenesis and hepatic lipogenesis.

## Author Contributions

**Chidimma Emmanuel Ibeneme:** investigation, writing – original draft, validation, writing – review and editing, data curation. **Item Justin Atangwho:** conceptualization, formal analysis, methodology, writing – review and editing, supervision. **Daniel Ejim Uti:** conceptualization, validation, writing – review and editing, formal analysis, software. **Uket Nta Obeten:** conceptualization, validation, writing – original draft, writing – review and editing, methodology. **Diana Ochuole Odey:** investigation, validation, data curation, writing – review and editing. **Emmanuel O. Ibeneme:** writing – review and editing, formal analysis. **Wilson Arong Obio:** investigation, writing – original draft, validation, writing – review and editing, data curation. **Godwin Eneji Egbung:** conceptualization, validation, writing – review and editing, supervision. **Chinedum Ekeleme Martins:** investigation, writing – original draft, validation, data curation, writing – review and editing. **Louisiane Patrick Nangah:** investigation, writing – original draft, validation, writing – review and editing, data curation.

## Funding

The authors have nothing to report.

## Ethics Statement

Animal experiments were reviewed and approved by the Faculty Animal Research Ethics Committee (FAREC‐FBMS), Faculty of Basic Medical Sciences, University of Calabar, Nigeria, with full ethical approval for the proposal titled “Molecular and in silico effects of Coula edulis nut extract on glucose metabolism in high fat diet obese Wistar rats.” Approval No.: 406BCM3225; Committee Ref.: FAREC/PA/UC/049; Date: 10/05/2023 (10 May 2023).

## Consent

The authors have nothing to report.

## Conflicts of Interest

The authors declare no conflicts of interest.

## Supporting information


**Table S1:** (a) Concentration and purity of liver RNA extracts. (b) Concentration and purity of adipose tissue RNA extracts.
**Table S2:** Effect of *Coula edulis* on histology of the adipose tissue sections of treatment groups vs controls. PLATE S1, Photomicrograph of Normal Control (Adipose tissue) (H&E X 400); FC, fat cell; PLATE S2, Photomicrograph of HFD + NT (Adipose tissue) (H&E X 400); HFD, high‐fat diet; NT, no treatment; PLATE S3, Photomicrograph of HFD + OR (Adipose tissue) (H&E X 400); OR, orlistat; PLATE S4, Photomicrograph of HFD + CE1g (Adipose tissue) (H&E X 400); CE, *Coula edulis*; PLATE S5, Photomicrograph of HFD + CE2g (Adipose tissue) (H&E X 400); PLATE S6, Photomicrograph of ND + NT (Adipose tissue) (H&E X 400); ND, normal diet; PLATE S7, Photomicrograph of ND + OR (Adipose tissue) (H&E X 400); PLATE S8, Photomicrograph of ND + CE1g (Adipose tissue) (H&E X 400); PLATE S9, Photomicrograph of ND + CE2g (Adipose tissue) (H&E X 400).
**Table S3:** (a) Summary/overview of the results obtained in this study—Part I. (b) Summary/overview of the results obtained in this study—Part II.

## Data Availability

The data that supports the findings of this study are available in Tables S1–S3 of this article.
