# Supplementary material for: Multi‐Target Anti‐Obesity Potential of Coula edulis Nut Extract in High‐Fat Diet‐Induced Obesity in Rats
Source: Food Sci Nutr. 2026 Aug 13;14(8):e72209. doi: 10.1002/fsn3.72209 (PMC13473704; doi:10.1002/fsn3.72209)
Supplement: Supplementary file 1 [file FSN3-14-e72209-s001.docx]

**TABLE S1a:	Concentration and purity of liver RNA extracts**

| Group | Conc. (ng/µL) | Purity |
| --- | --- | --- |
| NC | 1745.05±50.3 | 2.04±0 |
| HFD+NT | 808.15±46.3 | 2.0±0.05 |
| HFD+OR | 1235.9±30.2 | 1.98±0.04 |
| HFD+CE1g | 223.25±18.5 | 2.06±0.03 |
| HFD+CE2g | 1478.1±94.3 | 2.03±0.03 |
| ND+NT | 92.65±13.6 | 1.88±0.04 |
| ND+OR | 1934±101.3 | 2.03±0.02 |
| ND+CE1g | 535.45±50.5 | 2.01±0.09 |
| ND+CE2g | 131.1±20.3 | 2.0±0.05 |

Values are expressed as the mean ± SD, n = 6. NC= normal control, HFD = high-fat diet, ND = normal diet, OR = Orlistat, NT = no treatment CE = *Coula edulis*

**Table S1 b:	Concentration and purity of adipose tissue RNA extracts**

| Group | Conc. (ng/µL) | Purity |
| --- | --- | --- |
| Normal Control | 28.65±2.3 | 2.35±0.06 |
| HFD+NT | 42.7±9.6 | 2.11±0.07 |
| HFD+OR | 44.55±12.3 | 1.96±0.08 |
| HFD+CE1g | 71.55±7.6 | 1.94±0.1 |
| HFD+CE2g | 105.5±8.9 | 1.98±0.08 |
| ND+NT | 47.05±15.3 | 1.90±0.12 |
| ND+OR | 82.9±18.6 | 1.80±0.05 |
| ND+CE1g | 201.1±34.6 | 1.80±0.07 |
| ND+CE2g | 72.95±13.5 | 1.84±0.05 |

Values are expressed as the mean ± SD, n = 6. NC= normal control, HFD = high-fat diet, ND = normal diet, OR = Orlistat, NT = no treatment CE = *Coula edulis*

**TABLE S2: Effect of *Coula edulis* on histology of the adipose tissue sections of treatment groups vs controls**

| **Treatment groups** | **Observation** | **Inference** |
| --- | --- | --- |
| NC | Typical fat cells (FC) with regular collagenous fibres | Typical adipose tissue histology |
| HFD+NT | Hypertrophy of fat cell with increased fat accumulation was observed compared with normal control. Irregularly arranged collagenous fibers with increased presence of matured adipocytes was also observed | Fat cell expansion and increased presence of matured adipocyte indicates accumulation of fat, hence obesity |
| HFD+OR | Orlistat administration caused hypotrophy of fat cells and reduction in matured adipocyte number. | Reduction of fat accumulation showing anti-obesity effect |
| HFD+CE1g | Fat cells shrunk mildly and a slight reduction in number of adipocyte was observed compared with HFD+NT. | Slight reduction in fat accumulation |
| HFD+CE2g | Scattered scanty mature adipocyte was observed. Reduction in fat cell size was also observed compared with HFD+NT. | Attenuation of hypertrophy and hyperplasia of adipocyte, indicating anti-obesity effect. |
| ND+NT | Replacement of the high fat diet with normal diet in the obese rats reduced the number of adipocyte compared with HFD+NT. Also a reduction in adipocyte size and lipid droplet was observed | Gradual reversal of the  effect of high fat diet |
| ND+OR | Further decrease in size of fat cell and scattered scanty adipocyte was observed compared with ND+NT | Reduction in fat accumulation |
| ND+CE1g | Decreased adipocyte size and scattered scanty matured adipocyte observed | Slight reduction in lipid droplet. |
| ND+CE2g | Significant decrease in adipocyte size and scattered scanty matured adipocyte compared to ND+NT was observed | Anti-obesity effect |

NC= normal control, HFD = high-fat diet, ND = normal diet, OR = Orlistat, NT = no treatment CE = *Coula edulis*

PLATE S1- Photomicrograph of Normal Control (Adipose tissue) (H&E X 400). FC = fat cell

PLATE S2- Photomicrograph of HFD+NT (Adipose tissue) (H&E X 400). HFD = high-fat diet, NT = no treatment, FC = fat cell

PLATE S3- Photomicrograph of HFD+OR (Adipose tissue) (H&E X 400). HFD = high-fat diet, OR = orlistat, FC = fat cell

PLATE S4- Photomicrograph of HFD+CE1g (Adipose tissue) (H&E X 400). HFD = high-fat diet, CE = *Coula edulis,* FC = fat cell

PLATE S5- Photomicrograph of HFD+CE2g (Adipose tissue) (H&E X 400). HFD = high-fat diet, CE = *Coula edulis,* FC = fat cell.

PLATE S6- Photomicrograph of ND+NT (Adipose tissue) (H&E X 400). ND = normal diet, NT = no treatment, FC = fat cell

PLATE S7- Photomicrograph of ND+OR (Adipose tissue) (H&E X 400). ND = normal diet, OR = orlistat, FC = fat cell

PLATE S8- Photomicrograph of ND+CE1g (Adipose tissue) (H&E X 400). ND = normal diet, CE = *Coula edulis,* FC = fat cell

PLATE S9- Photomicrograph of ND+CE2g (Adipose tissue) (H&E X 400). ND = normal diet, CE = *Coula edulis,* FC = fat cell

**TABLE S3a: Summary/overview of the results obtained in this study – Part I**

| **S/N** | **Objective** | **Indicators measured** | **Results** | **Implication/Interpretation** |
| --- | --- | --- | --- | --- |
| 1. | Induction of experimental obesity | Diet intake  Calorie intake  Body weight  Abdominal circumference  Lee’s index  Body mass index | No change  ↑ Caloric intake  ↑ Bodyweight gain ≥ 20% of NC  ↑ *p* < 0.05 vs NC  ↑ *p* < 0.05 vs NC  ↑ *p* < 0.05 vs NC | Obesity induced |
| 2. | Intervention vs anthropometric indices | Diet intake  Calorie intake  Body weight gain  Abdominal circumference  Lee’s index  Body mass index | **Batch 1** \| **Batch 2**; No change \| No change; No change \| **↓** Intake vs ND+NT; **↓** *p* < 0.05 vs HFD+NT \| **↓** *p* < 0.05 vs ND+NT; **↓** *p* < 0.05 vs HFD+NT \| **↓** *p* < 0.05 vs ND+NT; **↓** *p* < 0.05 vs HFD+NT \| **↓** *p* < 0.05 vs ND+NT; **↓** *p* < 0.05 vs HFD+NT \| **↓** *p* < 0.05 vs ND+NT | Reduction in body weight gain Indicated |
| 3 | Intervention vs serum lipid profile and cardiovascular risk indicators | Triacylglycerol  Total cholestrol  LDL-c  VLDL-c  HDL-c  Atherogenic index  Atherogenic coefficient  Cardiovascular risk ratio | **Batch 1** \| **Batch 2**; **↓** *p* < 0.05 vs HFD+NT \| **↓** *p* < 0.05 vs ND+NT; **↓** *p* < 0.05 vs HFD+NT \| **↓** *p* < 0.05 vs ND+NT; **↓** *p* < 0.05 vs HFD+NT \| **↓** *p* < 0.05 vs ND+NT; **↓** *p* < 0.05 vs HFD+NT \| **↓** *p* < 0.05 vs ND+NT; **↑** *p* < 0.05 vs HFD+NT \| ↑ *p* < 0.05 vs ND+NT; **↓** *p* < 0.05 vs HFD+NT \| **↓** *p* < 0.05 vs ND+NT; **↓** *p* < 0.05 vs HFD+NT \| **↓** *p* < 0.05 vs ND+NT; **↓** *p* < 0.05 vs HFD+NT \| **↓** *p* < 0.05 vs ND+NT | Improved serum lipid profile and reduction in obesity-induced cardiovascular risk. |
| 4 | Intervention vs pancreatic lipase activity | Pancreatic lipase activity | **Batch1** \| **Batch 2**; **↓** *p* < 0.05 vs HFD+NT \| ↓ *p* < 0.05 vs ND+NT | Inhibition of fat digestion |
| 5 | Intervention vs tissue lipid storage | Triacylglycerol  Total cholesterol  Total lipids  Phospholipids  Tissue weight | **Batch 1** \| **Batch 2**; **↓** *p* < 0.05 vs HFD+NT \| **↓** *p* < 0.05 vs ND+NT; **↓** *p* < 0.05 vs HFD+NT \| **↓** *p* < 0.05 vs ND+NT; **↓** *p* < 0.05 vs HFD+NT \| **↓** *p* < 0.05 vs ND+NT; **↓** *p* < 0.05 vs HFD+NT \| **↓** *p* < 0.05 vs ND+NT; **↓** *p* < 0.05 vs HFD+NT \| ↓ *p* < 0.05 vs ND+NT | Reduction in tissue lipid storage |

Key: LDL-c – Low density lipoprotein cholesterol, VLDL-c – Very low density lipoprotein cholesterol, HDL-c – High density cholesterol, NC – Normal control

Batch 1: HFD+NT= High-fat diet fed group with no treatment, Batch 2: ND+NT= Normal diet fed group with no treatment.

**TABLE S3b: Summary/overview of the results obtained in this study – Part II**

| **S/N** | **Objective** | **Indicators measured** | **Results** | **Implication/interpretation** |
| --- | --- | --- | --- | --- |
| 1 | Intervention vs hepatic cholesterol biosynthesis | HMG-CoA reductase activity | **Batch1** \| **Batch 2**; **↓** *p* < 0.05 vs HFD+NT \| ↓ *p* < 0.05 vs ND+NT | Inhibition of hepatic cholesterol biosynthesis |
| 2 | Intervention vs oxidative stress indicators | SOD  GPx  GSH  MDA | **Batch 1** \| **Batch 2**; **↑** ***p*** < 0.05 vs HFD+NT \| **↑** *p* < 0.05 vs ND+NT; **↑** *p* < 0.05 vs HFD+NT \| **↑** ***p*** < 0.05 vs ND+NT; **↑** ***p*** < 0.05 vs HFD+NT \| **↑** ***p*** < 0.05 vs ND+NT; **↓** *p* < 0.05 vs HFD+NT \| **↓** *p* < 0.05 vs ND+NT | Reduction in oxidative stress and lipid peroxidation in the adipose tissue. |
| 3 | Intervention vs adipogenesis regulatory (upstream) genes. | PPARγ  C/EBP α  SREBP-1c | **Batch 1** \| **Batch 2**; **↓** *p* < 0.05 vs HFD+NT \| **↓** *p* < 0.05 vs ND+NT; **↓** *p* < 0.05 vs HFD+NT \| **↓** *p* < 0.05 vs ND+NT; **↓** *p* < 0.05 vs HFD+NT \| **↓** *p* < 0.05 vs ND+NT | Reduction in adipogenesis via downregulation of expression of regulatory genes. |

Key: HMG-CoA - 3-hydroxy-3-methylglutaryl coenzyme A, SOD – Superoxide dismutase, GPx – Glutathione peroxidase, GSH – Reduced glutathione, MDA – malondialdehyde, PPAP – Peroxisome proliferator-activated receptor, C/EBP – CCAAT/enhancer-binding protein, SREBP – Sterol regulatory-element binding protein,. Batch 1: HFD+NT= Hih fat diet with no treatment, Batch 2: ND+NT= Normal diet with no treatment
